# Cyclodextrin-Containing Tannic Acid–Lipoic Acid Hydrogels for the Loading and Reduction-Responsive Release of Nile Red

**DOI:** 10.3390/gels12100868

**Published:** 2026-09-25

**Authors:** Yuzheng Zhu, Kazuya U. Kobayashi, Akihiko Hatano, Manabu Suzuki, Kenichi Niikura

**Affiliations:** 1 Environmental Symbiotic System Major, Graduate School, Nippon Institute of Technology, Miyashiro 345-8501, Saitama, Japan; 2 Department of Mechanical Engineering, Nippon Institute of Technology, Miyashiro 345-8501, Saitama, Japan; 3 Department of Chemistry, Shibaura Institute of Technology, Fukasaku, Minumaku, Saitama-City 337-8570, Saitama, Japan; 4 Research & Development Center for Advanced Materials and Technology, Nippon Institute of Technology, Miyashiro 345-8501, Saitama, Japan

**Keywords:** cyclodextrin, lipoic acid, tannic acid hydrogel, Nile Red, hydrophobic cargo, encapsulation efficiency, reduction-responsive release

## Abstract

Hydrophobic molecules are generally difficult to incorporate into aqueous hydrogels because of their poor water solubility. In this study, a cyclodextrin (CD)-containing tannic acid–lipoic acid (TA–LA) hydrogel was developed using lipoic acid-modified β-cyclodextrin (LACD). LACD retained the host–guest recognition ability of the cyclodextrin cavity after lipoic acid modification and was successfully incorporated into the TA–LA hydrogel without interfering with gel formation. Using Nile Red (NR) as a model hydrophobic molecule, the LACD-containing hydrogel showed higher NR encapsulation efficiency and greater cyclodextrin retention than the hydrogel containing unmodified β-cyclodextrin. The LACD-containing hydrogel also exhibited reduction-responsive NR release in the presence of dithiothreitol (DTT). These results show that LACD can be incorporated into the TA–LA hydrogel while retaining the host–guest recognition ability of the cyclodextrin cavity and can be used for reduction-responsive release of NR.

## 1. Introduction

Hydrogels are functional materials that contain large amounts of water and can be used in the body. Hydrogels are suitable for biomedical applications, such as the release of drugs over time [1,2]. On the other hand, it is not easy to introduce and retain hydrophobic molecules inside a hydrophilic matrix. To overcome this issue, host molecules, such as cyclodextrin (CD), have been introduced into hydrogels [3,4,5,6]. CDs can improve the dispersion of hydrophobic molecules in water because they have a hydrophilic outer surface and a relatively hydrophobic cavity. Many hydrogels containing CDs have been reported. One of the most common methods is to chemically attach CDs to polymer side chains [7,8,9]. Another method is to encapsulate drugs in CDs and incorporate the CD–drug complexes into hydrogels through noncovalent interactions [10]. However, CDs that are simply dispersed in hydrogels may be lost during washing or drug release. Thus, in CD-containing hydrogels, not only the binding ability toward guest molecules but also the retention of CD within the matrix is an important issue. Hydrogels containing cyclodextrin derivatives via disulfide bonds have also been reported [8]. In these systems, drug release from the hydrogels in response to reducing agents, such as glutathione, has been reported.

α-Lipoic acid (LA, also known as thioctic acid) is a naturally derived 1,2-dithiolane compound whose cyclic disulfide bond can undergo ring-opening polymerization at 70 °C to form dynamic poly(disulfide)-related structures [11,12]. This property has been used to prepare injectable and self-healing LA-based hydrogels [13]. Chen et al. combined poly(lipoic acid) with tannic acid (TA) to prepare an injectable hydrogel originally named TATA [14]. To avoid confusion between the abbreviations of tannic acid and thioctic acid, this system is referred to here as the TA–LA hydrogel, where TA denotes tannic acid, and LA denotes lipoic acid. Later studies further expanded this platform. Shao et al. introduced phytic acid to improve shape stability, water-immersion stability, and wearable performance [15], while Tu et al. incorporated exosomes for sustained local delivery [16]. These studies show that the TA–LA hydrogel can be adapted for different functions, but the aqueous introduction and final encapsulation of poorly water-soluble small molecules in this system remain less studied. In this study, we extended the TA–LA hydrogel platform by incorporating LACD, thereby introducing cyclodextrin-based host–guest functionality into the hydrogel. Accordingly, 6-lipoylamido-β-cyclodextrin (LACD) was first synthesized (Scheme 1). LACD is a known compound, and its synthesis using different coupling methods has been reported [17,18]. LACD has also been used for the surface modification of gold nanoparticles, but to the best of our knowledge, its application to hydrogels has not been reported. LACD was associated with Nile Red (NR), a model hydrophobic compound, and then introduced into the TA–LA hydrogel.

Several methods have been reported for introducing hydrophobic molecules into hydrogels using CDs. As a simple method, the hydrogel was immersed in an aqueous solution of CD–hydrophobic cargo [19]. In this case, the introduction depends on the diffusion of the CD–hydrophobic cargo into the hydrogel. As another method, a CD–hydrophobic cargo complex was prepared before gelation and introduced into the hydrogel precursor [10,20]. This method is particularly useful for introducing poorly water-soluble cargo because the guest molecule can be introduced into the precursor as a complex with CD and does not need to diffuse into the hydrogel after gel formation.

The novelty of this study is the introduction of lipoic acid-modified cyclodextrin into a hydrogel composed of poly(lipoic acid) and tannic acid. The lipoic acid group introduced into CD can participate in hydrogel formation, which may help retain CD in the TA–LA network while maintaining its inclusion ability. In this design, hydrophobic molecules can be introduced into the hydrogel as CD–guest complexes during gel formation. In addition, the disulfide bonds in the TA–LA network may allow reduction-responsive degradation of the hydrogel and release of the cargo. Although the incorporation of disulfide-containing cyclodextrin derivatives into 2-hydroxyethyl methacrylate (HEMA)-based hydrogels has been reported [8], to the best of our knowledge, the use of lipoic acid-modified cyclodextrin in tannic acid–lipoic acid (TA–LA) hydrogels has not been reported.

The cavity of β-CD can encapsulate hydrophobic guest molecules, while the LA group may participate in ring-opening polymerization and/or disulfide exchange with LA-derived chains during gel formation, which may help retain LACD in the TA–LA network. For cargo loading, an LACD–NR host–guest complex was first prepared and then mixed with the components of the TA–LA hydrogel during gel formation. Unmodified β-CD, which does not have a reactive LA group, was used as a control for the physically introduced CD. By comparing LACD with β-CD, we aimed to investigate whether LA modification improves the retention of CD in the TA–LA network and enables reduction-responsive release of Nile Red dye. The LACD-based design presented in this study may provide a new approach for introducing poorly water-soluble molecules into hydrogels as CD–guest complexes, retaining them in the hydrogel network, and releasing them in response to reducing conditions. In addition, because this system is composed of tannic acid, lipoic acid, and cyclodextrin, it may have potential for biomedical applications, such as hydrogels for drug release.

## 2. Results and Discussion

### 2.1. Characterization of Synthetic LACD

As shown in Scheme 1, LACD was synthesized through amide coupling between lipoic acid and 6-amino-6-deoxy-cyclodextrin. We used nuclear magnetic resonance (NMR) spectroscopy and electrospray ionization high-resolution mass spectrometry (ESI-HRMS) to confirm the formation of 6-lipoylamido-β-cyclodextrin (LACD) (Figures S1–S3). The ^1^H NMR spectrum of LACD (Figure S1) showed that the amide proton (NH) appeared at 7.6 ppm. The typical signals of the β-CD skeleton were observed at 3.5–6 ppm, especially the clear anomeric H-1 signal at around 4.8 ppm. The signals in the 3.3–1.3 ppm region corresponded to the characteristic protons of the lipoic acid moiety. The integration ratios of these signals were consistent with the expected values for LACD and with those reported previously for LACD [17], although a different synthetic method was used in the present study. These results suggest that substantial amounts of unreacted starting materials were not present. In addition, the ^13^C NMR spectrum (Figure S2) showed the complete carbon signals of the β-CD skeleton at around 60–103 ppm, while the aliphatic carbon signals of the lipoic acid moiety appeared at 25–57 ppm. In particular, a clear carbonyl carbon signal was observed at around 172 ppm, further supporting the formation of an amide bond between lipoic acid and β-CD [21]. HRMS (Figure S3) showed a sodium adduction at *m*/*z* 1344.4076 ([M + Na]^+^), which agreed well with the calculated value (1344.4079), further confirming the successful synthesis of LACD. These three analytical results confirmed the successful synthesis of the target LACD as a slightly yellowish-white powder (81% yield).

### 2.2. Host–Guest Complexation Behavior of LACD Toward Nile Red

#### 2.2.1. Aqueous Incorporation of Nile Red by LACD

To examine whether LACD retained the host–guest inclusion ability of β-cyclodextrin, we investigated the interaction between LACD and Nile Red (NR) in aqueous solution. Cyclodextrin inclusion complexes are commonly prepared by solution-based methods and characterized by UV–Vis and FTIR spectroscopy. NR is a hydrophobic dye that is almost insoluble in water under ambient conditions, and its UV–Vis absorption in water is also barely detectable. Inclusion by β-CD has been shown to improve the aqueous solubility and absorbance signal of NR [22].

As shown in Figure 1, NR showed almost no detectable absorbance near 575 nm in pure water, with a value of approximately 0.003. The corresponding UV–Vis absorption spectra (500–650 nm) are provided in Figure S4. In contrast, when the same amount of NR was added to 0.2–1.0 mM LACD solutions, the absorbance increased to 0.05–0.08. Even at the lowest LACD concentration of 0.2 mM, the absorbance of NR was clearly higher than that in pure water.

This change showed a trend similar to that reported by Ray et al. for the β-CD–NR system [22]. Because NR has very low solubility in water, the marked increase in absorbance in the presence of LACD suggested that the aqueous state of NR has been clearly improved through the β-CD cavity. However, the increase in absorbance alone could not exclude other possible factors. Therefore, in Section 2.2.2, 1-Adamantanecarboxylic acid (ADA) was used as a competitive guest to further examine whether this phenomenon was related to host–guest interactions involving the cyclodextrin cavity.

#### 2.2.2. Competitive Effect of ADA on the LACD–NR System

Adamantane salts and their derivatives have strong host–guest affinity for β-cyclodextrin, with binding constants reaching the order of 10^5^ M^−1^. They are therefore commonly used as model guests for CD cavity recognition and competitive binding [23,24]. As shown in Figure 2, the absorbance of NR decreased clearly after the addition of ADA at all tested LACD concentrations. The corresponding UV–Vis absorption spectra (500–650 nm) are provided in Figure S5. This decrease in absorbance is considered to be due to the release of NR from the CD cavity by competitive binding of ADA, followed by aggregation of NR in water [22]. The ADA-induced absorbance decrease was largest in the 0.2 mM LACD group at approximately 71% and decreased to about 42% in the 1.0 mM group (Figure S6). This may be because more CD cavities remained available at higher LACD concentrations, so the same amount of ADA caused a smaller decrease in the NR signal. These results indicate that the β-CD cavity participated in the LACD–NR interaction and contributed to the enhanced aqueous absorbance of NR.

#### 2.2.3. FTIR Analysis of the LACD–NR Inclusion Complex

Because attenuation or disappearance of guest-specific IR bands has been reported for cyclodextrin inclusion complexes [25,26], we examined the FTIR spectra of the dried NR/β-CD and NR/LACD inclusion complexes. Although the absolute amounts of the samples used for the FTIR measurements differed, the molar ratio of NR to CD was kept constant at 1:8 for both samples. As shown in Figure 3, NR displayed characteristic bands at 1622, 1494, and 780 cm^−1^, with the 1622 cm^−1^ band. These results are consistent with the reported characteristic absorption bands of Nile Red in the region around 1550–1620 cm^−1^ [27]. The complete FTIR spectra are shown in Figure S7. LACD showed a broad O–H band at 3306 cm^−1^, amide-related bands at 1653 and 1583 cm^−1^, and β-CD skeletal bands at 1157 and 1031 cm^−1^ [25,28]. The physical mixture largely retained the spectral features of both components. In the dried LACD–NR inclusion complex, the characteristic NR bands at 1494 and 780 cm^−1^ were markedly attenuated or no longer separately resolved. The accompanying shift in the broad O–H band from 3306 to 3387 cm^−1^ provided additional support for changes in the local interaction environment. The β-CD skeletal bands at 1157 and 1030 cm^−1^ remained detectable. These changes support the formation of the LACD–NR inclusion complex rather than a simple physical mixture. Moreover, the results show that the LACD–NR-associated state was retained in the dried material subsequently used for hydrogel preparation.

### 2.3. Hydrogel Formation and Component Retention Behavior

#### 2.3.1. Hydrogel Formation, Morphology, and Rheological Properties

Previous studies on the original TATA hydrogel, referred to as the TA–LA hydrogel in this study, and related systems have shown that the TA–LA system can form a supramolecular hydrogel through ring-opening polymerization of LA and multiple noncovalent and dynamic interactions with TA [14]. To adapt the reported TA–LA gelation procedure to CD-containing formulations, the preparation conditions were modified from those used by Chen et al. In the original method [14], the TA/LA/Tris mixture was heated at 70 °C for 60 s and then subjected to one freeze–thaw cycle. In the present study, the TA0.3 formulation (Table 1) was heated at 75 °C for 10 min, and β-CD or LACD was added together with LA before heating. Thus, the CD component was introduced during formation of the TA–LA hydrogel rather than being added to a preformed gel.

As shown in Figure S8, TA–LA, β-CD0.1, and LACD0.1 all formed hydrogels that maintained their own shape, indicating that the introduction of β-CD or LACD at the tested concentrations did not disrupt the macroscopic gelation ability of the TA–LA system. In this study, SEM was further used to compare the surface morphologies of freeze-dried TA–LA, β-CD0.1, and LACD0.1 hydrogels.

The SEM images of the freeze-dried samples showed that TA–LA had a relatively smooth and continuous surface (Figure 4a), β-CD0.1 displayed localized protrusions and crater-like structures (Figure 4b), whereas LACD0.1 had a rougher surface with folds and small pore-like structures (Figure 4c). These observations indicate that β-CD and LACD did not markedly affect macroscopic gel formation but produced different effects on the freeze-dried morphology.

We performed strain- and frequency-sweep tests on the TA–LA, β-CD0.1, and LACD0.1 hydrogels (Figure 5). In the strain-sweep tests, the storage modulus (G′) remained higher than the loss modulus (G″) over a broad strain range for all three hydrogels, showing that their elastic response was dominant (Figure 5a). At 1% strain, LACD0.1 showed the highest G′, followed by TA–LA and β-CD0.1. The frequency-sweep tests showed the same trend. At a fixed strain of 0.3%, G′ remained higher than G″ for all three hydrogels over the angular-frequency range of 0.628–628 rad s^−1^ (Figure 5b). LACD0.1 showed the highest G′ throughout the measured frequency range, whereas the G′ of β-CD0.1 was lower than that of TA–LA over most of the frequency range. Both tests demonstrated that the incorporation of LACD increased the stiffness of the hydrogel network, whereas native β-CD may have slightly softened the TA–LA network. β-CD was mainly physically dispersed within the hydrogel. Its relatively large cyclic structure and hydrophilic surface may have altered the local arrangement and hydrogen-bond distribution within the TA–LA network, thereby interfering to some extent with network formation [29]. In contrast, the LA moiety of LACD may have been incorporated into the LA-derived network, thereby increasing the stiffness of the hydrogel network.

#### 2.3.2. Polymerization-Related ^1^H NMR Analysis

Gel formation in Section 2.3.1 does not clarify whether the CD components affect the relative proportions of residual LA monomer and poly(LA)-related species. Therefore, the freeze-dried TA–LA, β-CD0.1, and LACD0.1 hydrogels were dissolved in DMSO-*d*_6_ and analyzed by ^1^H NMR (Figure 6). The full ^1^H NMR spectrum is provided in Figures S9 and S10. The original TATA study proposed that LA ring-opening polymerization and multiple interactions between LA and TA are important for gel formation in this system [14]. Because this system forms a dynamically crosslinked network rather than a linear polymer with well-defined end groups, the absolute degree of polymerization was not calculated. Instead, the poly(LA)-related molar fraction was compared using the Mono-LA signal at around 2.4 ppm and the poly(LA)-related signal at around 2.8 ppm, assigned with reference to the reported ^1^H NMR spectra of poly(α-lipoic acid) [30].

As shown in Table 2, the poly(LA)-related molar fraction of β-CD0.1 was lower than that of TA–LA, whereas that of LACD0.1 was higher (the calculation of the poly(LA)-related molar fraction is described in Equation (1)). These results suggest that native β-CD interfered with LA polymerization in the hydrogel, resulting in a higher relative proportion of residual LA monomer. In contrast, LACD did not inhibit the formation of poly(LA)-related species, as indicated by the absence of an increase in Mono-LA-related species in LACD0.1. Therefore, the effect of LACD on the TA–LA system is unlikely to be attributed to inhibition of LA polymerization.

#### 2.3.3. Retention of CD-Containing Components After Washing

We further compared the retention behavior of β-CD and LACD in the TA–LA hydrogels and analyzed the samples by ^1^H NMR after water immersion and rinsing. As shown in Figure 7, the CD H-1 signal at approximately 4.8 ppm was markedly weakened in the β-CD hydrogel after washing, whereas the corresponding signal remained clearly visible in the LACD hydrogel (all spectra were normalized to the poly(LA)-related signal at 3.00). The full ^1^H NMR spectrum is provided in Figure S11. The relative CD H-1 signal remaining after washing was calculated according to Equation (2). The β-CD0.1 hydrogel retained only 21% of the relative CD H-1 signal after washing, whereas the corresponding value for LACD0.1 was 72% (Table 3). The same trend was also observed for the LACD0.2 and β-CD0.2 hydrogels (Figures S12 and S13). These results show that native β-CD was more readily lost from the TA–LA hydrogel during immersion and washing, whereas LACD was retained more effectively. Together with the higher storage modulus of LACD0.1 observed in Section 2.3.1 and its higher poly(LA)-related molar fraction in Section 2.3.2, the greater retention of LACD supports the possibility that its LA moiety participated in the formation of the LA-derived network, thereby anchoring LACD more effectively within the hydrogel.

To evaluate the retention of solid mass after washing, we compared the dry mass ratio and gel fraction of each group. The dry mass ratios before washing were approximately 48–53% for all groups (Table S1), indicating similar overall solid contents in the initial wet hydrogels. The gel mass fraction was calculated according to Equation (S1). After washing, the dry mass ratios decreased markedly in all groups, showing that some soluble or unretained components were removed during the washing process. A significant overall difference was observed among the five groups, with TA–LA and LACD0.1 both showing significantly higher values than β-CD0.2. At both formulation levels, the gel fractions of LACD0.1 and LACD0.2 were approximately 13 percentage points higher than those of the corresponding β-CD formulations. This trend suggests that LACD-containing hydrogels were more stable during washing and is consistent with the results described in Section 2.3.2.

Gel fraction reflects the overall solid fraction retained in the hydrogel, whereas ^1^H NMR more directly reflects the relative change in CD-derived signals. Taken together, these results indicate that LACD-derived CD components were relatively better retained in the TA–LA matrix than native β-CD. This may be related to the LA moiety in LACD, which could improve its compatibility with the TA–LA network.

### 2.4. Wet-Mass Changes and DTT-Induced Degradation

The original TATA study evaluated the conventional swelling behavior of freeze-dried TA–LA hydrogels and reported a maximum swelling ratio of approximately 800% [14]. In the present study, freshly prepared wet hydrogels were used directly to evaluate their mass changes in PB in the absence or presence of DTT, under the same conditions used in the release experiments.

TA–LAs with different TA concentrations (TA0.2, TA0.3, and TA0.4) were first compared in PB based on their wet-mass behavior. The mass change ratio was calculated according to Equation (3). TA0.2 showed a markedly smaller mass increase, whereas TA0.4 exhibited rapid early mass gain followed by a subsequent decrease, suggesting that water uptake occurred together with partial component loss or network relaxation. In contrast, TA0.3 showed a more stable mass increase without obvious mass loss. The three formulations differed significantly at 120 min; therefore, TA0.3 was selected as the base formulation for the subsequent CD-containing hydrogels (Figure 8a).

After the addition of LACD or β-CD to TA0.3, the early mass increase of the hydrogels in PB was markedly accelerated (Figure 8b). Because both types of CD showed a similar trend, this change may be related to the hydrophilic hydroxyl groups in the CD structure. After approximately 20–30 min, the mass of some CD-containing hydrogels began to decrease, indicating that water uptake and the loss of soluble or unretained components occurred simultaneously. Although both the 0.1 and 0.2 formulations formed self-supporting hydrogels, the 0.2 formulations showed larger early mass changes and a more pronounced subsequent decline in PB. Therefore, β-CD0.1 and LACD0.1, which showed more moderate wet-mass changes while maintaining stable gel formation, were selected as the matrices for subsequent NR loading experiments.

Under DTT conditions, all groups showed an initial mass increase followed by a gradual decrease, and the decline occurred earlier in 0.1 M DTT than in 0.01 M DTT (Figure 8c,d). This is consistent with the combined effects of water uptake and the reductive disruption of the LA/poly(disulfide)-related network by DTT [8,31]. Among these groups, the LACD-containing hydrogels showed slower mass loss, indicating that the LACD groups were overall less readily degraded under reductive DTT conditions. The differences in water uptake and DTT-induced mass changes after CD addition may be associated with differences in the hydrogel network structure. The ^1^H NMR results showed different relative distributions of residual LA monomer and poly(LA)-related species among the formulations. In particular, the higher poly(LA)-related fraction of LACD0.1 was consistent with its higher storage modulus (G′), suggesting a comparatively stronger network. The SEM observations provide additional morphological information, although the freeze-dried structures may not fully represent the hydrogels in the hydrated state.

### 2.5. Cargo Loading Efficiency

This study used NR as a hydrophobic model cargo and adopted a pre-association-assisted loading strategy, in which CD/LACD–NR complexes were formed first and then incorporated during TA–LA hydrogel formation. Preformed CD–guest complexes have been introduced into hydrogels to improve the dispersion and stability of poorly water-soluble cargos. Similar strategies, in which hydrophobic molecules are first encapsulated in CDs and subsequently incorporated into hydrogels, have also been reported [32,33].

Before gel formation, the absorbance at 575 nm of NR (3.3 μg/mL, 10.4 μM) mixed with 0.8 mM LACD was higher than that of the β-CD system at the same concentration, indicating that LACD enabled more NR to remain in water (Table 4). The corresponding averaged UV–Vis absorption spectra are provided in Figure S14. This result is consistent with the LACD–NR solubilization observed in Section 2.2.

After gel formation, the hydrogels were completely degraded by DTT, and the incorporated NR was quantified after redissolution in methanol (Figure S15). Based on the initial NR feed and the gel portion used for analysis, the theoretical NR amount corresponding to 200 mg of hydrogel was 33.3 μg. The encapsulation efficiency (EE) was calculated according to Equation (4). The NR encapsulation efficiency of the CD-free TA–LA hydrogel was only approximately 6%, indicating that hydrophobic NR was difficult to introduce into and retain within the TA–LA matrix in the absence of a CD host. The value increased markedly after the addition of β-CD, while LACD0.1 reached 77%, which was higher than the 57% obtained for β-CD0.1 (Table 4).

The higher NR encapsulation efficiency of LACD0.1 was mainly attributed to the more effective introduction of NR into water before gel formation. At the same concentration, the LACD–NR system showed a higher absorbance at 575 nm than the β-CD–NR system, and the ADA competition experiment supported the involvement of the CD cavity of LACD in NR interaction. Therefore, LACD allowed more NR-containing species to remain in water and subsequently be incorporated into the TA–LA hydrogel during gel formation. This indicates that the LACD-assisted pre-association was more favorable than native β-CD for the initial loading of hydrophobic NR.

### 2.6. Release Behavior Under PB Condition in the Absence or Presence of DTT

Before gel formation, the CD/LACD–NR solutions prepared in deionized water showed an absorption maximum at approximately 575 nm, whereas the hydrogel release media collected in PB showed a maximum at approximately 595 nm. This shift was likely caused mainly by the presence of TA. In addition, the presence of TA improved the apparent solubility or dispersion of NR in water, regardless of the presence of CD. The absorption spectra of NR added to water containing TA with or without β-CD are shown in Figure S16. When TA was added to an aqueous solution containing the NR–CD complex, the absorption maximum of NR showed a red shift. Furthermore, when NR was directly added to an aqueous solution containing TA alone, the solution remained colored, and the NR absorption band showed not only a red shift but also an increase in absorbance. This increase in absorbance is consistent with improved dispersion or apparent solubility of NR in the aqueous TA solution. In this study, NR was preloaded into LACD or β-CD before gel formation. This preloading provides a strategy for incorporating hydrophobic NR into the hydrogel during gel formation, while TA may also contribute to the dispersion of NR within the hydrogel.

In addition, a portion of the release medium collected at each time point was evaporated to dryness, and the recovered NR was redissolved in methanol. The cumulative release amount was then calculated using the calibration curve (see experimental Section 4.9). The cumulative NR release was calculated according to Equation (S2). At the end of the release experiment, the hydrogel was completely decomposed by treatment with excess DTT and heating. After removal of the solvent, the remaining NR was dissolved in methanol and quantified. The total amount of NR was defined as the sum of the NR released into the release medium at each sampling time and the NR remaining in the hydrogel. This total amount was defined as 100% and used as the reference for calculating the cumulative NR release.

Because the CD-free TA–LA hydrogel showed an NR encapsulation efficiency of only approximately 6%, a reliable comparative release profile could not be obtained. Therefore, the release study focused on comparing the β-CD0.1 and LACD0.1 hydrogels. In PB, the β-CD hydrogel showed higher absorbance in water and a higher early cumulative release than the LACD hydrogel (Figure 9a,b). The corresponding averaged UV–Vis absorption spectra are provided in Figures S17 and S18. Although LACD0.1 showed a higher NR encapsulation efficiency than β-CD0.1 in Section 2.5, its aqueous absorbance in PB increased more slowly at early times and remained lower overall. Therefore, the lower PB signal cannot be explained by a lower initial NR loading but instead suggests stronger retention of NR-containing species within the LACD hydrogel. Together with the better retention of LACD-derived CD signals after washing observed in Section 2.3, this result suggests that LACD and its associated NR species may be more strongly restricted within the network, whereas native β-CD-associated species may enter the water more readily. Because NR can readily aggregate and lose its detectable absorption in water after leaving the CD cavity, a lower absorbance does not necessarily indicate the absence of release. The methanolic redissolution results confirmed that NR was continuously released from the LACD hydrogel, although its early release rate was lower than that of the β-CD hydrogel. Similar retardation of early release through host–cargo interactions with network-retained CD hosts has also been reported in another CD-containing hydrogel [8].

The DTT release experiments were designed to evaluate the reductive responsiveness of the selected LACD0.1 formulation. Under DTT conditions, both absorbance and cumulative release increased more rapidly, with 0.1 M DTT producing a stronger effect than 0.01 M DTT (Figure 10a,b). Consistent with these release profiles, both blank and NR-loaded LACD0.1 hydrogels progressively degraded during exposure to 0.1 M DTT from 0 to 3 h (Figure 10c). This result is consistent with the DTT-induced wet-mass decrease observed in Section 2.4. This suggests that the disulfide-containing network of the TA–LA hydrogel is disrupted under reductive conditions, promoting cargo release [11,14,34].

In both DTT groups, the aqueous absorbance decreased after reaching a maximum, whereas the cumulative release measured after methanol redissolution continued to increase. Therefore, the decrease in absorbance did not represent a cessation of release. As shown by the dilution experiment in Section S9, the absorbance of LACD–NR decreased similarly in PB and DTT after dilution, mainly because of changes in the LACD–NR equilibrium rather than a specific effect of DTT (Figures S19–S23).

In PB, the LACD hydrogel released NR more slowly than the β-CD hydrogel during 0.5–3 h, with the release rate (zero-order rate constant, k, in Table 5) decreasing from 8.2 to 5.9% h^−1^, or by about 28%. In contrast, DTT accelerated release from the LACD hydrogel in a concentration-dependent manner, increasing the rate to 10.7% h^−1^ at 0.01 M and 14.3% h^−1^ at 0.1 M. The 24 h cumulative release also increased from approximately 54% in PB to 66% and 71% in 0.01 and 0.1 M DTT, respectively. These results show that LACD slowed early release compared with native β-CD, while retaining a clear DTT concentration-dependent release response. Korsmeyer–Peppas fitting yielded *n* values of 0.79–0.94 (Table 5 and Figure S24 for the original fitting plots), suggesting that release was governed by both diffusion and network changes [35] rather than by disulfide bond cleavage alone.

## 3. Conclusions

Lipoic acid-modified β-cyclodextrin (LACD) was successfully synthesized and subsequently incorporated into the TA–LA hydrogel without interfering with gel formation. Compared with the hydrogel containing native β-CD, the LACD-containing hydrogel exhibited a higher storage modulus and greater retention of the CD component after washing. Together with the increased poly(LA)-related molar fraction observed by ^1^H NMR, these findings suggest that the LA moiety of LACD participated in, or interacted more effectively with, the LA-derived network, thereby improving its retention within the hydrogel. UV–Vis analysis and the ADA competition experiment demonstrated that LACD retained the host–guest recognition ability of the CD cavity after LA modification. Consequently, LACD increased the aqueous solubility of NR before gel formation and, using the pre-inclusion-assisted gelation method, improved NR encapsulation efficiency to 77%, compared with 57% for the β-CD0.1 hydrogel and approximately 6% for the CD-free TA–LA hydrogel.

Release studies showed that the LACD0.1 hydrogel exhibited slower initial NR release than the β-CD0.1 hydrogel under physiological conditions, whereas both hydrogels reached similar cumulative release after 24 h. Under reducing conditions in the presence of DTT, both the initial release rate and cumulative release increased, consistent with disruption of the poly(LA)-derived network. Korsmeyer–Peppas analysis further suggested that NR release was governed by both diffusion and structural changes in the hydrogel network.

Overall, LACD functioned as a multifunctional CD derivative that improved the aqueous incorporation and encapsulation of Nile Red while enhancing CD retention within the TA–LA hydrogel. This molecular design provides a simple strategy for improving CD retention and enabling reduction-responsive release of Nile Red in polyphenol-based hydrogels. The present results suggest that this approach may also be applicable to the incorporation and release of other poorly water-soluble molecules.

## 4. Materials and Methods

### 4.1. Materials

Nile Red (>98.0%), 1-adamantanecarboxylic acid (>98.0%), DL-α-lipoic acid (>99.0%), 6-amino-6-deoxy-β-cyclodextrin (>95.0%), 4-(4,6-Dimethoxy-1,3,5-triazin-2-yl)-4-methylmorpholinium Chloride (DMTMM) (>95.0%), and DL-dithiothreitol (DTT, >98.0%) were purchased from Tokyo Chemical Industry Co., Ltd. (TCI, Tokyo, Japan). β-Cyclodextrin, tannic acid, methanol (≥99.8%), *N*, *N*-dimethylformamide (DMF, ≥99.5%), dichloromethane (≥99.0%), and triethylamine (TEA) were obtained from FUJIFILM Wako Pure Chemical Corporation (Osaka, Japan). Tris (hydroxymethyl) aminomethane and 0.1 mol/L phosphate buffer solution (PB, pH 7.4) were purchased from NACALAI TESQUE, INC. (Kyoto, Japan). Purified water was purchased from AS ONE Corporation (Osaka, Japan).

### 4.2. Instruments

UV–visible absorbance spectra were recorded with a UV-2600 spectrophotometer (Shimadzu Corporation, Kyoto, Japan). NMR spectra were acquired on an ECS-400 system (JEOL Ltd., Tokyo, Japan, 400 MHz). HRMS analysis was carried out using an Orbitrap Exploris 120 LC–quadrupole/Orbitrap mass spectrometer (Thermo Fisher Scientific, Waltham, MA, USA) equipped with an electrospray ionization (ESI) source at the Center for Analytical Instrumentation, Chiba University (the sample was prepared in DMF). FTIR spectra were recorded using a JASCO FT/IR-4600 spectrometer (JASCO Corporation, Tokyo, Japan) from 4000 to 400 cm^−1^ at a resolution of 4 cm^−1^ using 32 scans. SEM images were acquired using a Hitachi S-4800 FE-SEM (Hitachi High-Tech Corporation, Tokyo, Japan). The viscoelastic properties were measured using an MCR 303 rotational rheometer (Anton Paar GmbH, Graz, Austria).

### 4.3. Synthesis of LACD

DL-lipoic acid (72 mg, 0.35 mmol) and DMTMM (89 mg, 0.32 mmol) were weighed using an analytical balance, placed in a 10 mL screw-cap glass vial, and dissolved in DMF (4 mL). The solution was magnetically stirred for 20 min using a magnetic stirrer(RCN-3, EYELA, Tokyo Rikakikai Co., Ltd., Tokyo, Japan) at a stirring speed of approximately 250 rpm. Triethylamine (36 mg, 0.36 mmol) was then added, followed by 6-amino-6-deoxy-β-cyclodextrin (6-NH_2_-β-CD; 200 mg, 0.18 mmol). The mixture was continuously stirred at room temperature for 48 h under the same stirring conditions. We concentrated the reaction mixture under reduced pressure using a rotary evaporator connected to an oil-sealed rotary vacuum pump at a bath temperature of 45 °C, a rotation speed of 200 rpm for approximately 2 h to give a viscous residue. After evaporation, approximately 10 mL of pure water was added to the residue to obtain a cloudy suspension. The suspension was divided into ten microtubes and centrifuged at 10,600× *g* (12,000 rpm) for 15 min. The supernatant was removed, and the precipitate was dried. The dried precipitate was then washed with excess dichloromethane. The solid was collected by vacuum filtration through filter paper and dried under reduced pressure to give a slightly yellowish-white powder (193 mg, 81% yield). ^1^H NMR (400 MHz, DMSO-*d*_6_): δ 7.62 (s, 1H); 5.8–5.63 (m, 14H, OH); 4.88–4.80 (m, 7H); 4.56–4.40 (m, 6H, OH); 3.76–3.56 (m, 28H); 3.4–3.2 (m, 15H); 3.2–3.1 (m, 2H); 2.47 (m, 1H); 2.10 (m, 2H); 1.89 (m, 1H); 1.7–1.43 (m, 4H); 1.32–1.29 (m, 2H). ^13^C NMR (101 MHz, DMSO-*d*_6_): δ 172.4, 102.0, 81.6, 73.0, 72.5, 72.0, 59.8, 56.1, 38.1, 34.9, 34.0, 28.2, 24.8. HRMS (ESI) m/z: [M + Na]^+^ calculated for C_50_H_83_NNaO_35_S_2_^+^: 1344.4079; found 1344.4076.

### 4.4. Preparation and Evaluation of LACD–NR Inclusion Complexes

The LACD–NR inclusion complex was prepared by mixing LACD and NR in aqueous solution, followed by removal of the solvent. We prepared LACD solutions at concentrations of 0.2, 0.4, 0.6, 0.8, and 1.0 mM using 2 mL of deionized water. The solutions were heated to 75 °C until LACD dissolved and then cooled to room temperature. Nile Red (NR) methanol solution (10 μL, 1.26 mM) was added dropwise while vortex mixing at 2800 rpm using a Vortex-Genie 2 Digital mixer (Scientific Industries, Bohemia, NY, USA). A control sample containing deionized water and the same amount of NR solution was prepared in the same way. The samples were heated at 75 °C for 15 min to reduce the methanol content, followed by sonication in a water bath for 15 min using a Branson 1510 ultrasonic cleaner (Yamato Scientific Co., Ltd., Tokyo, Japan) at an initial bath temperature of approximately 20 °C to promote LACD–NR complex formation. UV–Vis absorption spectra were recorded in the range of 500 to 650 nm using a UV-2600 spectrophotometer (Shimadzu, Kyoto, Japan). The same instrument was used for all subsequent UV–Vis measurements. The absorbance values were baseline-corrected by setting the absorbance at 650 nm to zero. LACD blank solutions of corresponding concentrations were used as baseline references.

### 4.5. Competitive Displacement Assay

The competitive displacement assay used excess 1-adamantanecarboxylic acid (ADA) as the competing guest. The powder of ADA (2 mg, corresponding to a nominal concentration of 5.5 mM in 2 mL water) was added to each LACD–NR solution (0.2–1.0 mM) prepared as described above. The samples were mixed by magnetic stirring and then centrifuged at 3000 rpm for 10 min to remove undissolved ADA. We collected the supernatant and recorded its UV–Vis spectrum from 500 to 650 nm. The absorbance values were baseline-corrected by setting the absorbance at 650 nm to zero. ADA-treated LACD solutions without NR were processed under the same conditions, and their supernatants were used as the baseline references.

### 4.6. FTIR Analysis

The LACD–NR inclusion complex used for FTIR analysis was prepared using the same procedure with LACD (5.0 mg, 3.79 μmol) and NR (0.15 mg, 0.47 μmol), giving an LACD/NR molar ratio of approximately 8:1. The physical mixture was prepared by thoroughly mixing LACD (100 mg) and NR (3 mg) at the same molar ratio. Pure NR, pure LACD, the physical mixture, and the inclusion complex were dried under vacuum for 24 h, ground with spectroscopic-grade KBr, and compressed into transparent pellets. FTIR spectra were recorded using a JASCO FT/IR-4600 spectrometer (JASCO Corporation, Tokyo, Japan) from 4000 to 400 cm^−1^ at a resolution of 4 cm^−1^ using 32 scans, and the comparison spectra are shown over 4000–500 cm^−1^.

### 4.7. Preparation and Structural Analysis of CD-Containing TA–LA Hydrogels

#### 4.7.1. Preparation of CD-Containing TA–LA Hydrogels

Blank TA–LA hydrogels were prepared based on the previously reported tannic acid–thioctic acid hydrogel method [14]. We dissolved Tris base (300 mg, 2.48 mmol) and tannic acid (TA; 300 mg, 0.18 mmol) in 2 mL of deionized water in a glass vial and then added α-lipoic acid (LA; 1 g, 4.85 mmol). The mixture was vortex-mixed at 2800 rpm for 1 min and then heated in a water bath (SB-1300, Tokyo Rikakikai Co., Ltd., Tokyo, Japan) at 75 °C for 10 min. The mixture was subsequently placed in a freezer at −20 °C for at least 1 h. All subsequently prepared hydrogels were subjected to the same freezing treatment. In this formulation, the masses of Tris and TA each corresponded to 0.3 times the mass of LA. The same procedure gave TA0.2 and TA0.4 hydrogels by changing the amounts of Tris and TA, as shown in Table 1 (Section 2.3.1).

LACD-containing hydrogels followed the TA0.3 formulation. We added LACD together with LA at LACD/LA mass ratios of 0.1 and 0.2, giving LACD0.1 and LACD0.2 hydrogels. For the β-CD controls, we replaced LACD with β-CD under the same preparation conditions.

NR-loaded hydrogels were prepared using a pre-complexation-assisted loading route. We first dissolved LACD (30 mg, 0.023 mmol) or β-CD (30 mg, 0.028 mmol) in deionized water (30 mL) in a 100 mL round-bottom flask at 75 °C. For the CD-free TA–LA control, deionized water (30 mL) without CD was used instead. NR methanol solution (1.26 mM, 250 μL in total) was added dropwise to each solution in five portions during vortex mixing at 2800 rpm. The mixture was heated in an open vessel in a water bath at 75 °C for 15 min to reduce the methanol content, followed by sonication in a water bath at 40 kHz for 15 min. After centrifugation at approximately 664 ×g (3000 rpm) for 15 min, the supernatant was collected and evaporated to dryness by rotary evaporation at a bath temperature of 40 °C, a rotation speed of 200 rpm, and a pressure of 5 mmHg for approximately 120 min. The dried residue was then transferred to a 10 mL glass sample vial, to which Tris base (90 mg, 0.74 mmol), TA (90 mg, 0.05 mmol), LA (300 mg, 1.46 mmol), and deionized water (800 μL) were added. The mixture was mixed and heated at 75 °C for 10 min to form the NR-loaded hydrogel. The formulations of the NR-loaded hydrogels are summarized in Table S2.

#### 4.7.2. SEM Observation and Rheological Characterization

We observed the surface morphology of freeze-dried hydrogels by field-emission scanning electron microscopy (FE-SEM). TA–LA, β-CD0.1, and LACD0.1 hydrogel samples were freeze-dried for 48 h, mounted on SEM stubs, and sputter-coated with gold before observation. SEM images were acquired using a Hitachi S-4800 FE-SEM at an accelerating voltage of 5.0 kV and a working distance of 10.4 mm using the SE(U) detector. Images were recorded at magnifications of 300×, 5000×, and 20,000×. Representative images obtained at 5000× are shown in the main text.

A rotational rheometer ((MCR 303, Anton Paar GmbH, Graz, Austria)) with a 25 mm parallel plate geometry was used to evaluate the rheological properties of the hydrogels at 23 °C. A 1.5 g batch of each hydrogel formulation was prepared for the measurements, and the measuring gap was fixed at 1.0 mm. Strain sweeps were conducted from 0.01 to 1000% strain at a fixed angular frequency of 6.3 rad s^−1^. Frequency sweeps were conducted from 0.628 to 628 rad s^−1^ at a fixed strain of 0.3%. The storage modulus (G′) and loss modulus (G″) were recorded.

#### 4.7.3. ^1^H NMR Analysis, CD Retention and Gel Fraction

We dissolved freeze-dried TA–LA, β-CD0.1, and LACD0.1 hydrogels in DMSO-*d*_6_ (20 mg in 0.75 mL) and analyzed them by ^1^H NMR spectroscopy. The poly(LA) molar fraction was calculated from the integrated areas of the characteristic Mono-LA and poly(LA)-related signals after correction for the number of protons represented by each signal:

(1) $$\mathrm{Poly}(\mathrm{LA})\;\mathrm{molar}\;\mathrm{fraction}\;(\%)=\frac{I_{\mathrm{poly}}/3}{I_{\mathrm{Mono}}+I_{\mathrm{poly}}/3}\times 100$$

where $I_{\mathrm{Mono}}$ and $I_{\mathrm{poly}}$ represent the integrated areas of the Mono-LA and poly(LA) signals, respectively. The Mono-LA and poly(LA) signals correspond to 1H and 3H, respectively.

For the washing experiment, we cut TA–LA, LACD0.1, LACD0.2, β-CD0.1, and β-CD0.2 hydrogels into 100 mg pieces. Samples were immersed in deionized water (5 mL) for 24 h, rinsed with deionized water and ethanol, and then freeze-dried. The corresponding before-washing samples were freeze-dried directly without washing. We dissolved the dried LACD0.1, LACD0.2, β-CD0.1, and β-CD0.2 samples in DMSO-*d*_6_ (20 mg in 0.75 mL) and analyzed them by ^1^H NMR spectroscopy. The CD H-1 signal was normalized to a selected poly(LA)-related reference signal, and the relative CD H-1 signal remaining after washing was calculated as follows:

(2) $$\mathrm{Relative}\;\mathrm{CD}\;H\text{-}1\;\mathrm{signal}\;\mathrm{remaining}\;(\%)=\frac{{\left(I_{\mathrm{CD}}/I_{\mathrm{ref}}\right)}_{\mathrm{after}}}{{\left(I_{\mathrm{CD}}/I_{\mathrm{ref}}\right)}_{\mathrm{before}}}\times 100$$

where $I_{\mathrm{CD}}$ represents the integral of the CD H-1 signal, and $I_{\mathrm{ref}}$ represents the integral of the selected poly(LA)-related reference signal.

### 4.8. Wet-Mass Changes and DTT-Induced Degradation of TA–LA Hydrogels

Wet-mass changes were monitored using freshly prepared hydrogels without prior drying. The samples were immersed in PB or DTT solution to track changes in the mass of the preformed gels and assess DTT-induced degradation under conditions similar to those used in the release experiments. We immersed blank hydrogels with TA/LA mass ratios of 0.2, 0.3, and 0.4 relative to LA (150 mg each) in 5 mL of 0.1 M PB (pH 7.4). At 5, 10, 20, 30, 60, 90, and 120 min, each hydrogel was taken out, gently blotted to remove surface water, weighed, and returned to the original solution.

We separately prepared LACD hydrogels with LACD/LA mass ratios of 0, 0.1, and 0.2 and β-CD hydrogels with β-CD/LA mass ratios of 0.1 and 0.2 (150 mg each). To compare their wet-mass change in PB, each formulation was immersed in 5 mL of 0.1 M PB (pH 7.4), and the sample mass was recorded at the same time points using the same procedure.

For the DTT response experiment, LACD hydrogels with LACD/LA mass ratios of 0, 0.1, and 0.2 (150 mg each) were immersed in 5 mL of 0.01 or 0.1 M DTT in PB (0.1 M phosphate, pH 7.4). The mass change of each sample was recorded at 5, 10, 20, 30, 60, 90, and 120 min using the same procedure.

The mass change ratio (MCR) was calculated as follows:

(3) $$\mathrm{MCR}\;(\%)=\frac{W_{t}-W_{0}}{W_{0}}\times 100$$

where *W*_0_ is the initial hydrogel mass before immersion, and *W_t_* is the measured mass at time *t*.

### 4.9. Nile Red Incorporation and Release Studies

NR–LACD hydrogel samples (200 mg) were immersed in 2 mL of PB (0.1 M phosphate, pH 7.4) containing 0.1 or 0.01 M DTT, or in PB without DTT, and maintained under static conditions at room temperature (approximately 20 °C). As a control, NR–β-CD hydrogel samples (200 mg) were immersed in PB (pH 7.4) under the same static conditions at room temperature (approximately 20 °C). At 0.5, 1, 2, 3, 6, and 24 h, 200 μL of the release medium was withdrawn and replaced with the same volume of fresh medium. The absorbance of each sample was measured directly at 595 nm to monitor the NR-related optical signal in the aqueous release medium. Release media (200 μL) collected at the corresponding time points from composition-matched blank hydrogels prepared without NR and incubated in the same medium were used as baseline references. The collected aliquots were then evaporated to dryness, redissolved in 1 mL of methanol, and analyzed by UV–Vis spectroscopy. After 24 h, we added excess DTT to the remaining hydrogel and the final release medium, and the mixture was heated at 70 °C until the hydrogel completely dissolved. The solvent was evaporated, and the residue was redissolved in 2 mL of methanol for UV–Vis analysis. The NR amount in each methanol solution was calculated using the NR calibration curve in methanol, A = 0.0208C (Equation (S3)), where A is the absorbance, and C is the NR concentration in μg/mL. The corresponding averaged UV–Vis absorption spectra and calibration data are provided in Figures S25 and S26 and Table S3.

To calculate cumulative release, we corrected for repeated sampling and normalized the released amount to the total recoverable NR. Full equations are provided in Section S10 of the Supplementary Materials.

For NR encapsulation efficiency, we used NR-loaded CD-free TA–LA, NR–βCD, and NR–LACD hydrogel samples (200 mg each). We added excess DTT to each sample and heated the mixture at 70 °C until the hydrogel completely dissolved. After complete dissolution, the solvent was evaporated, and the residue was redissolved in 2 mL of methanol.

Encapsulation efficiency (EE) was calculated using the following:

(4) $$\mathrm{EE}\;(\%)=\frac{m_{\mathrm{measured}}}{m_{\mathrm{fed}}}\times 100$$

where *m_fed_* is the added amount of NR fed into each 200 mg hydrogel sample, and *m_measured_* is the total amount of NR recovered from the hydrogel sample after complete degradation and methanol extraction.

### 4.10. Data Analysis

All quantitative data are presented as mean ± standard deviation (SD) from three independent measurements unless otherwise stated. Early-stage release data over the first 3 h were fitted using the zero-order model, $CR\;(\mathrm{cumulative}\;\mathrm{release})=k_{0}t+b$, where $k_{0}$ represents the apparent release rate. Release data up to approximately 60% cumulative release were also fitted using the Korsmeyer–Peppas model, $M_{t}/M_{\infty }=k_{KP}t^{n}$, after logarithmic transformation. The goodness of fit was evaluated using $R^{2}$. The detailed fitting procedure, equations, and fitting plots for the Korsmeyer–Peppas model are provided in Section S11 of the Supporting Information.

## Figures and Tables

**Figure 1:**
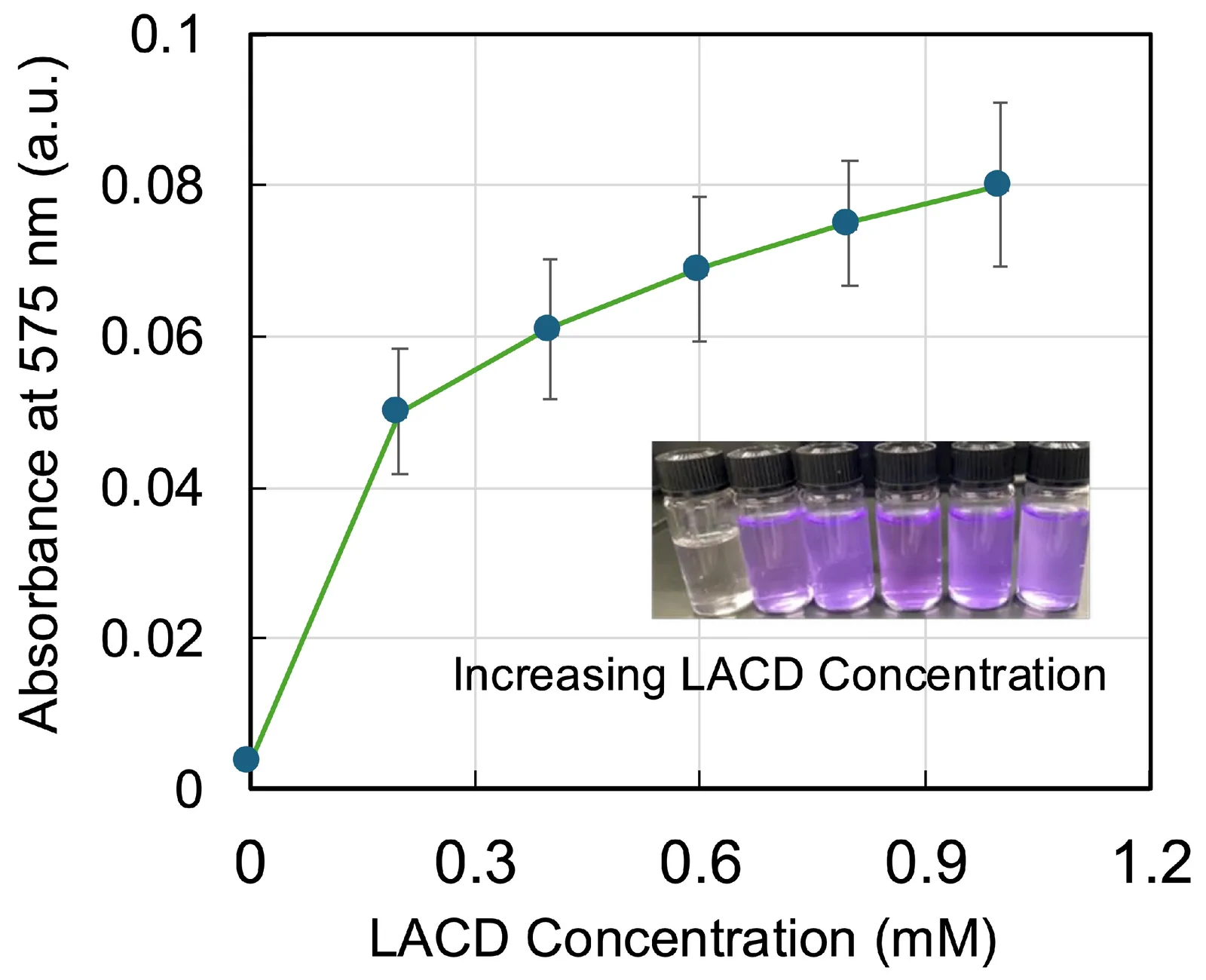
Absorbance at 575 nm of Nile Red in water containing different concentrations of LACD and a fixed amount of NR (2 μg/mL). Data are presented as mean ± SD (*n* = 3).

**Figure 2:**
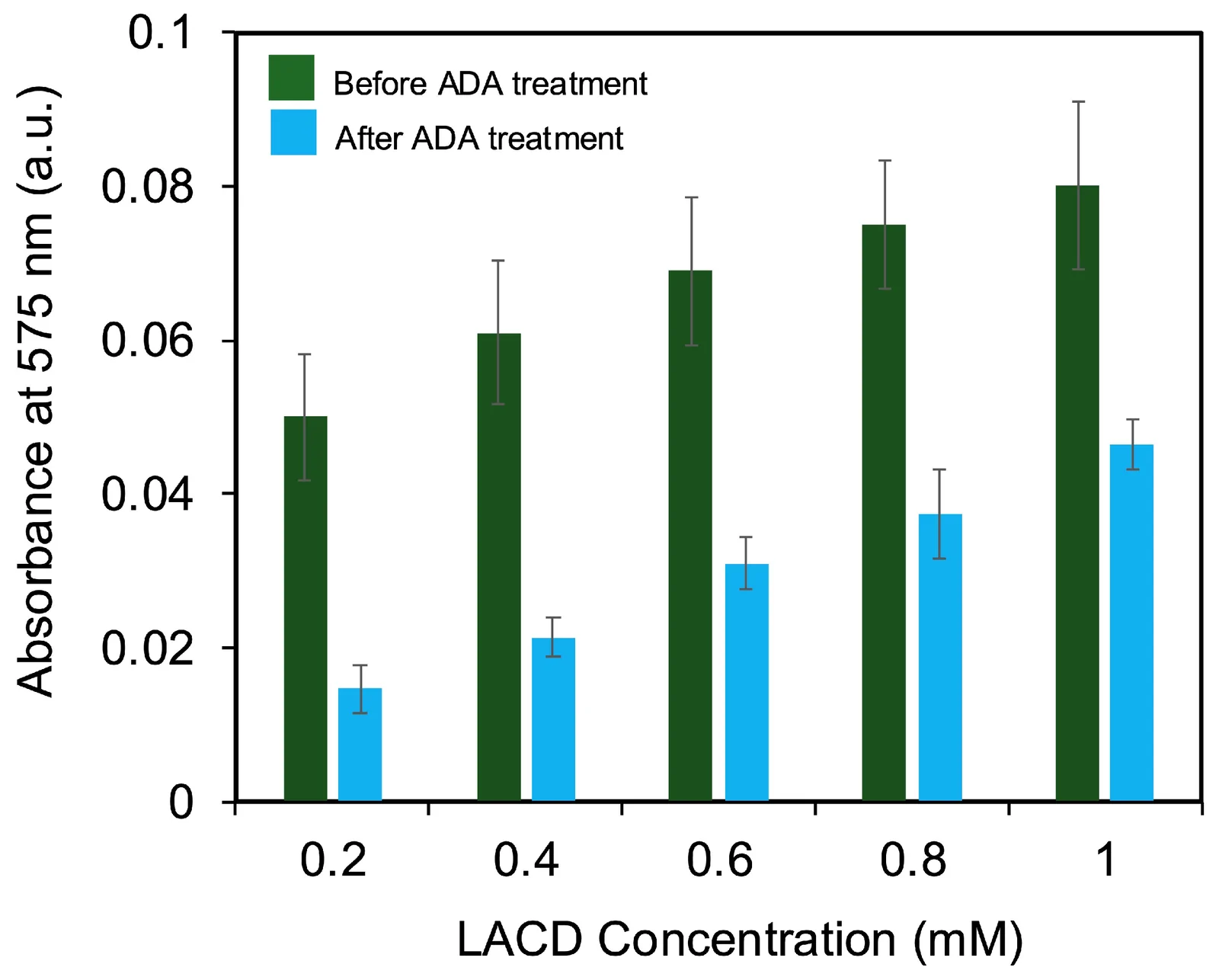
Absorbance at 575 nm of LACD–NR solutions at different LACD concentrations before and after ADA (1 mg/mL) addition. Data are presented as mean ± SD (*n* = 3).

**Figure 3:**
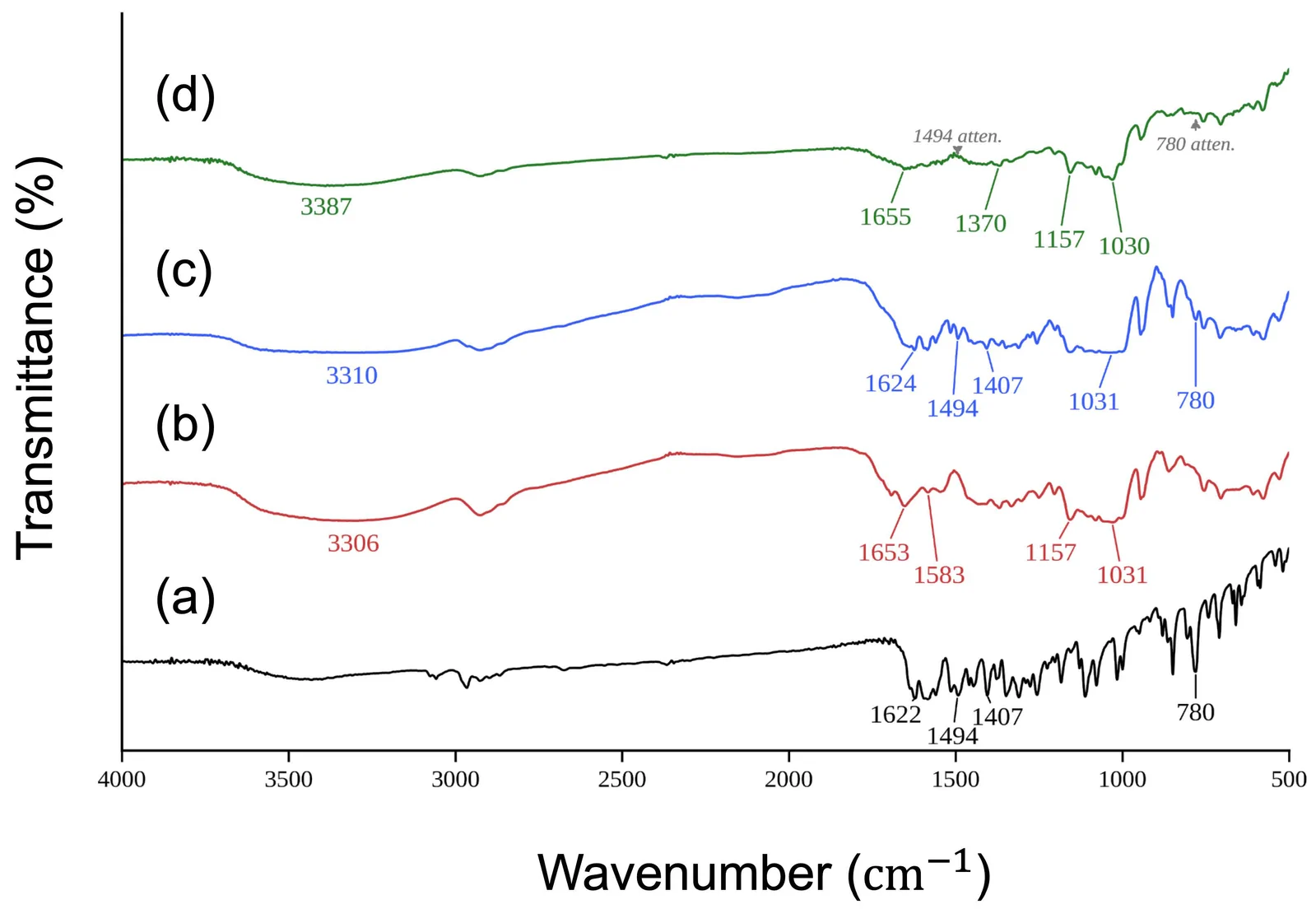
FTIR spectra: (**a**) NR, (**b**) LACD, (**c**) physical mixture of LACD and NR, and (**d**) LACD–NR inclusion complex. The LACD/NR host–guest molar ratio was 8:1 in both (**c**) and (**d**).

**Figure 4:**
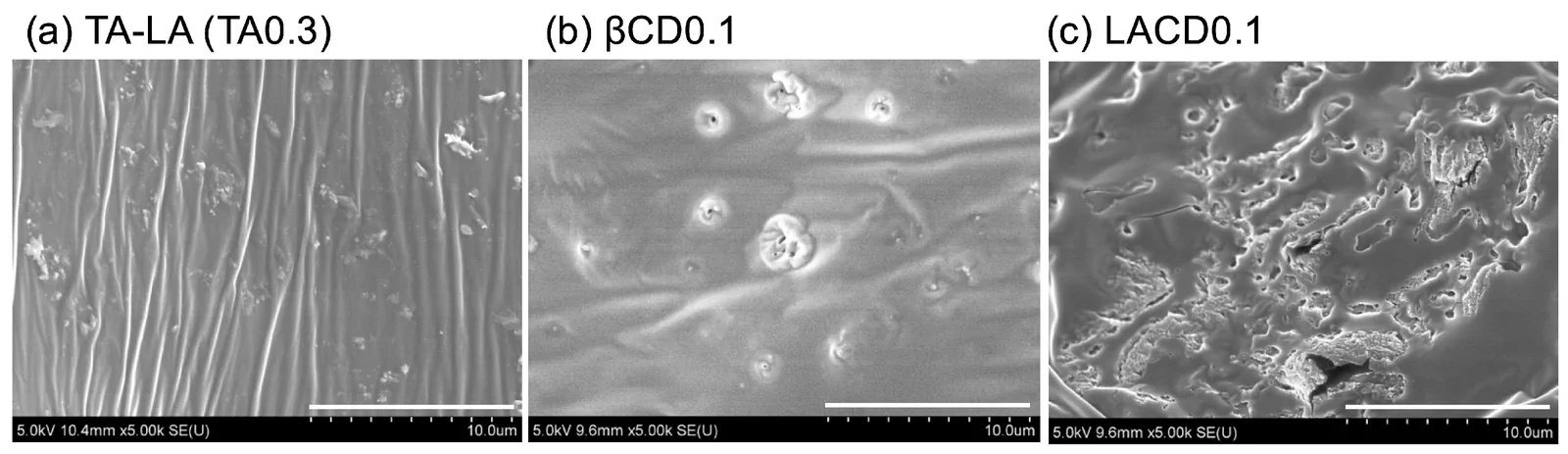
Macroscopic appearance and freeze-dried morphology of TA–LA (**a**), β-CD0.1 (**b**), and LACD0.1 (**c**) hydrogels. SEM images at 5000× magnification. Scale bar = 10 μm.

**Figure 5:**
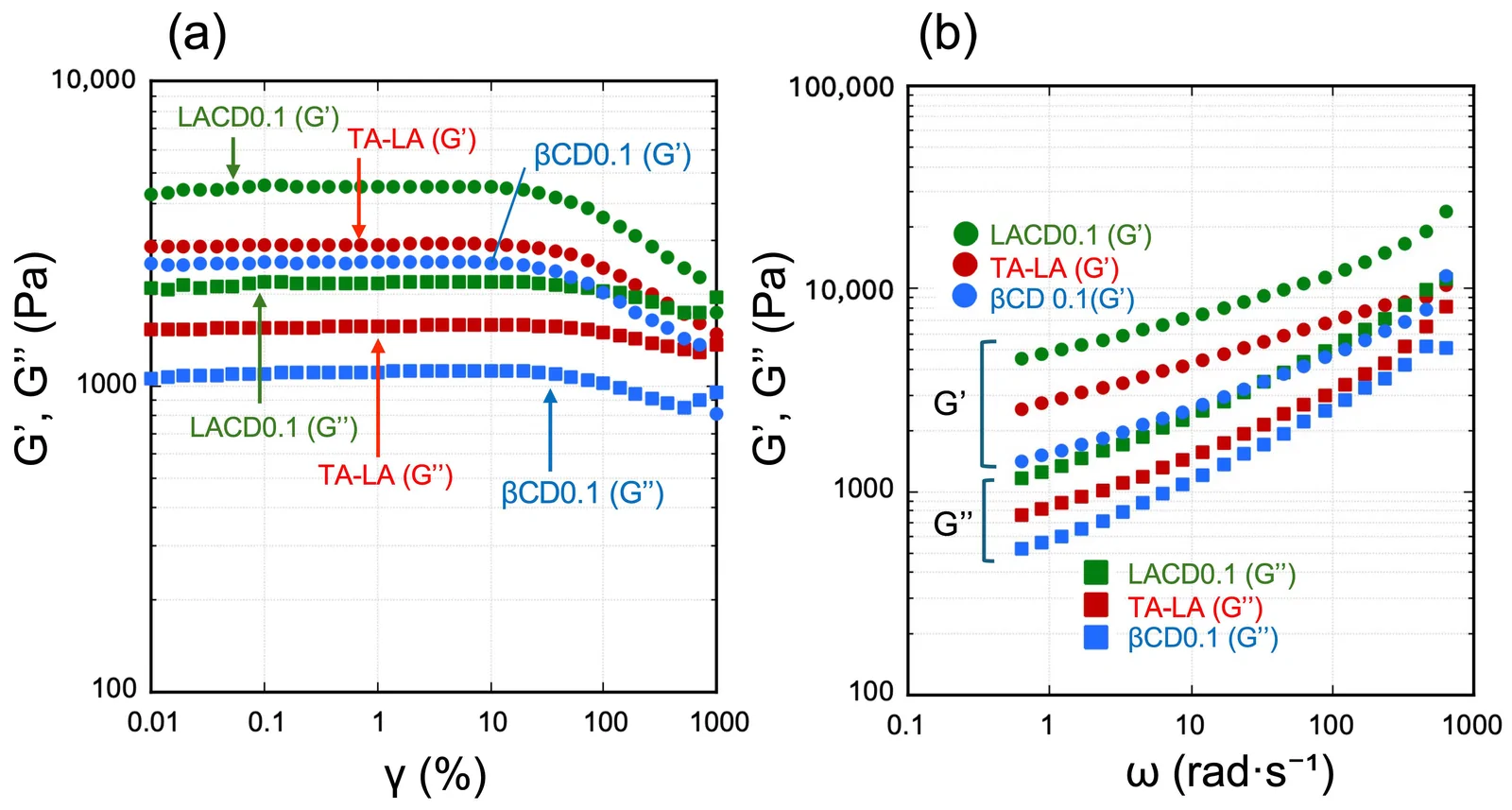
Rheological properties of TA–LA, LACD0.1, and β-CD0.1 hydrogels at 23 °C. (**a**) Strain-sweep measurements of the storage modulus (G′) and loss modulus (G″) at a fixed angular frequency of 6.3 rad s^−1^; (**b**) frequency-sweep measurements of G′ and G″ at a fixed strain of 0.3%.

**Figure 6:**
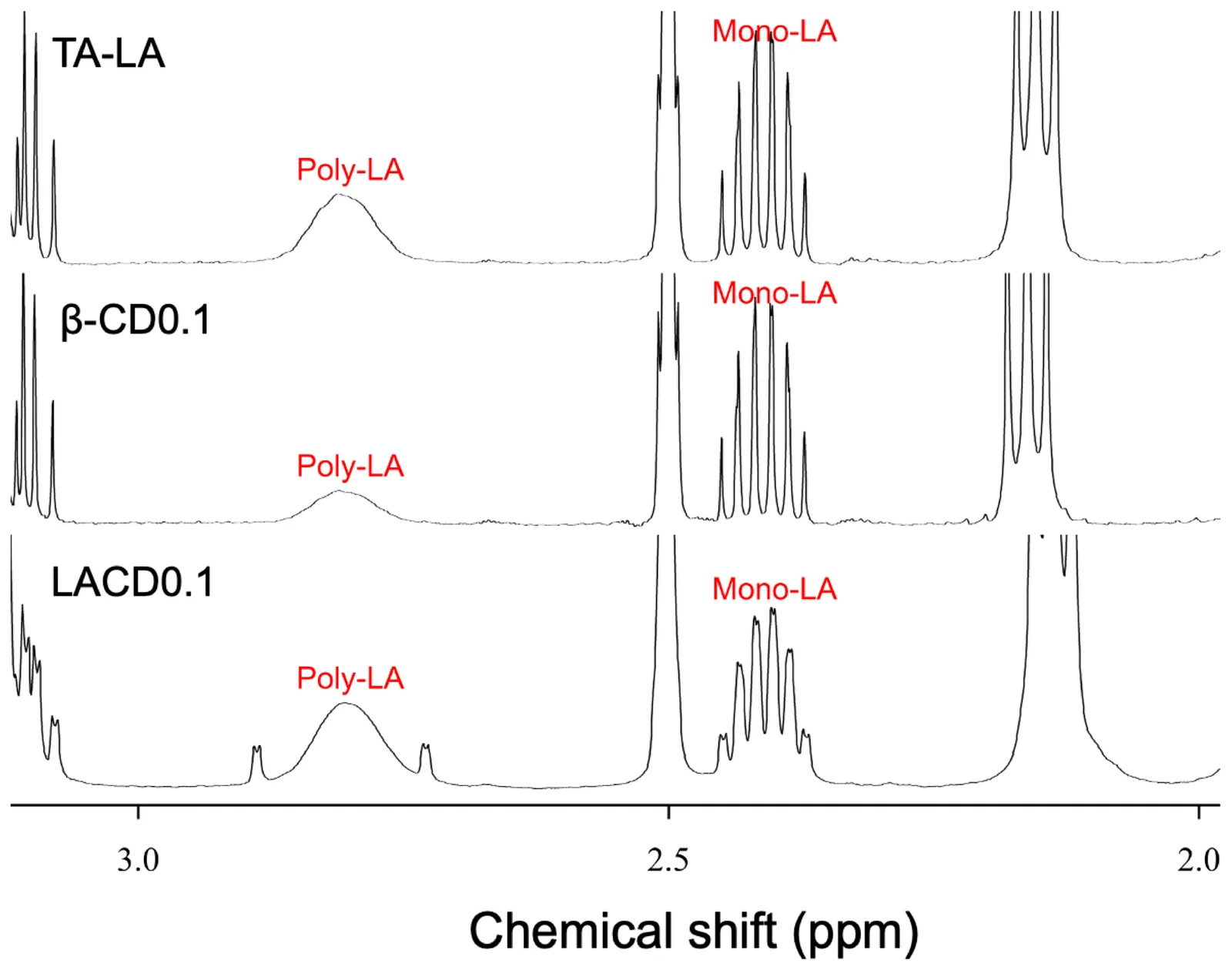
Typical polymerization-related ^1^H NMR signal comparison of TA–LA, β-CD0.1, and LACD0.1 hydrogels in DMSO-*d*_6_ (400 MHz). The spectra are shown in the 3.1–1.9 ppm region.

**Figure 7:**
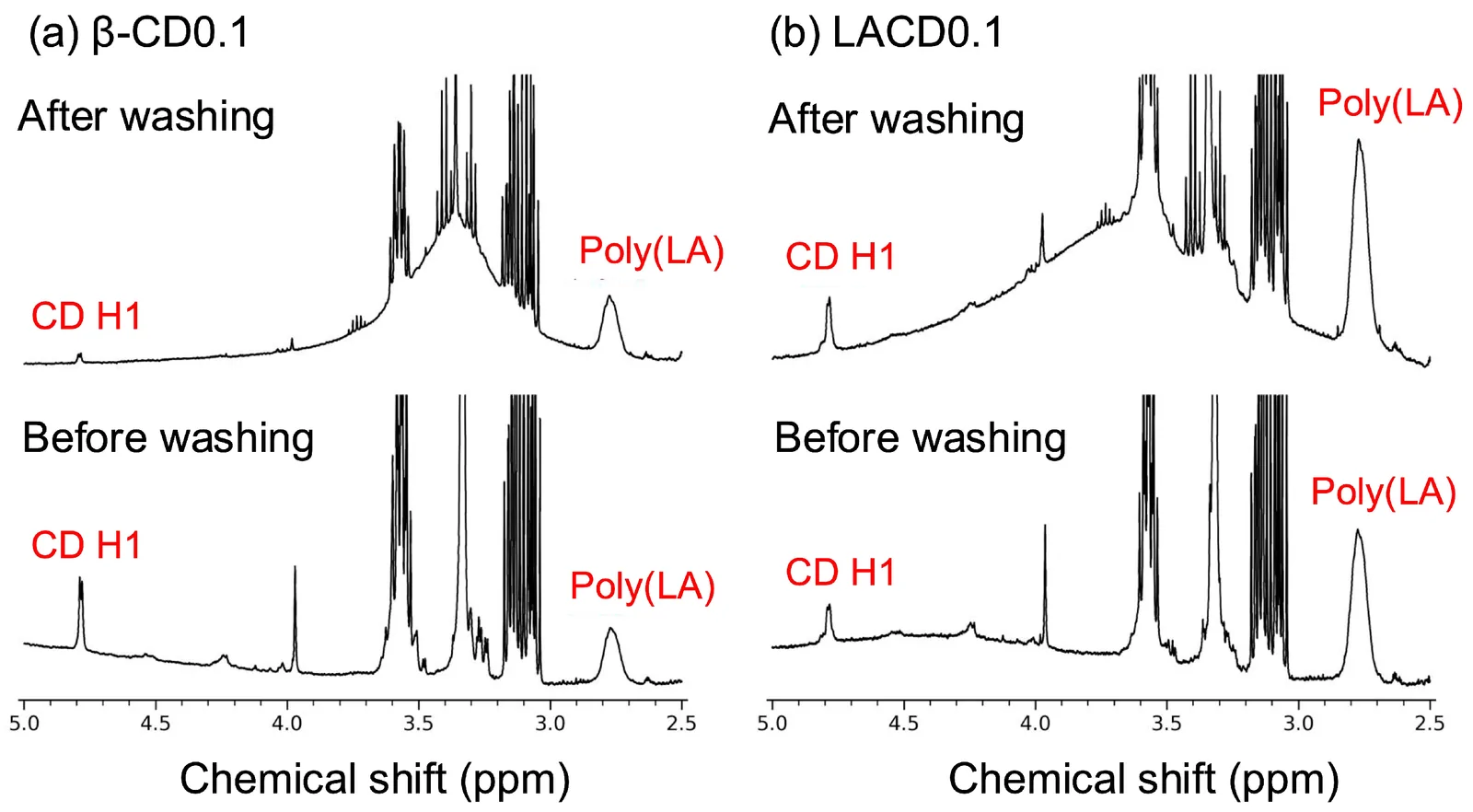
^1^H NMR spectra (2.5–5.0 ppm) of CD-containing TA–LA hydrogels before and after washing with water in DMSO-*d*_6_ (400 MHz): (**a**) β-CD0.1, (**b**) LACD0.1.

**Figure 8:**
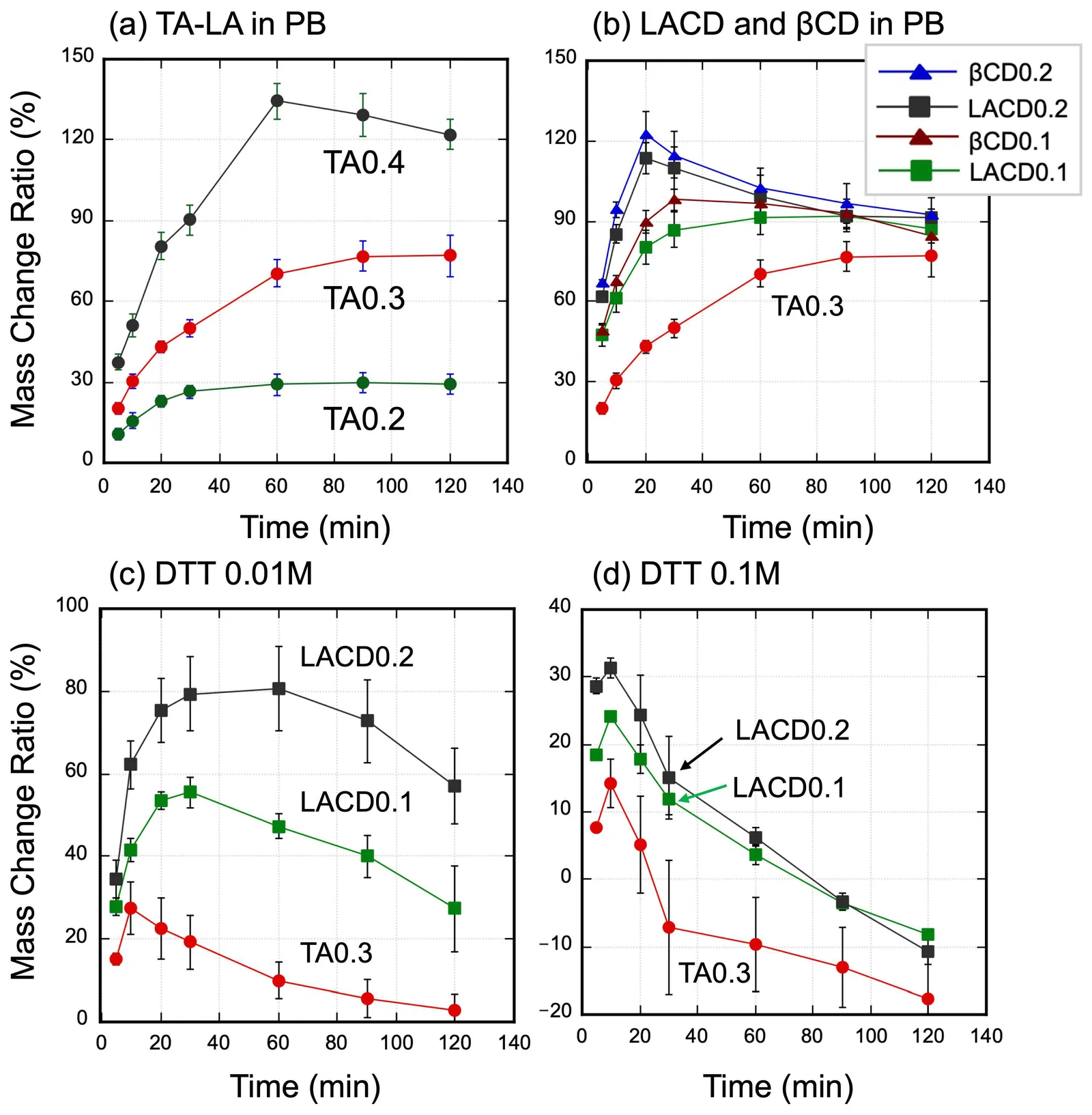
Wet-mass changes of TA–LA and CD-containing TA–LA hydrogels under PB and DTT conditions over 2 h. (**a**) TA–LA gels containing TA0.2, TA0.3, and TA0.4 hydrogels in 0.1 M PB (pH 7.4); (**b**) TA0.3 (same as in (**a**)), LACD0.1, LACD0.2, β-CD0.1, and β-CD0.2 hydrogels in 0.1 M PB (pH 7.4); (**c**) TA0.3, LACD0.1, and LACD0.2 hydrogels in 0.01 M DTT; and (**d**) TA0.3, LACD0.1, and LACD0.2 hydrogels in 0.1 M DTT. Data are presented as mean ± SD (*n* = 3).

**Figure 9:**
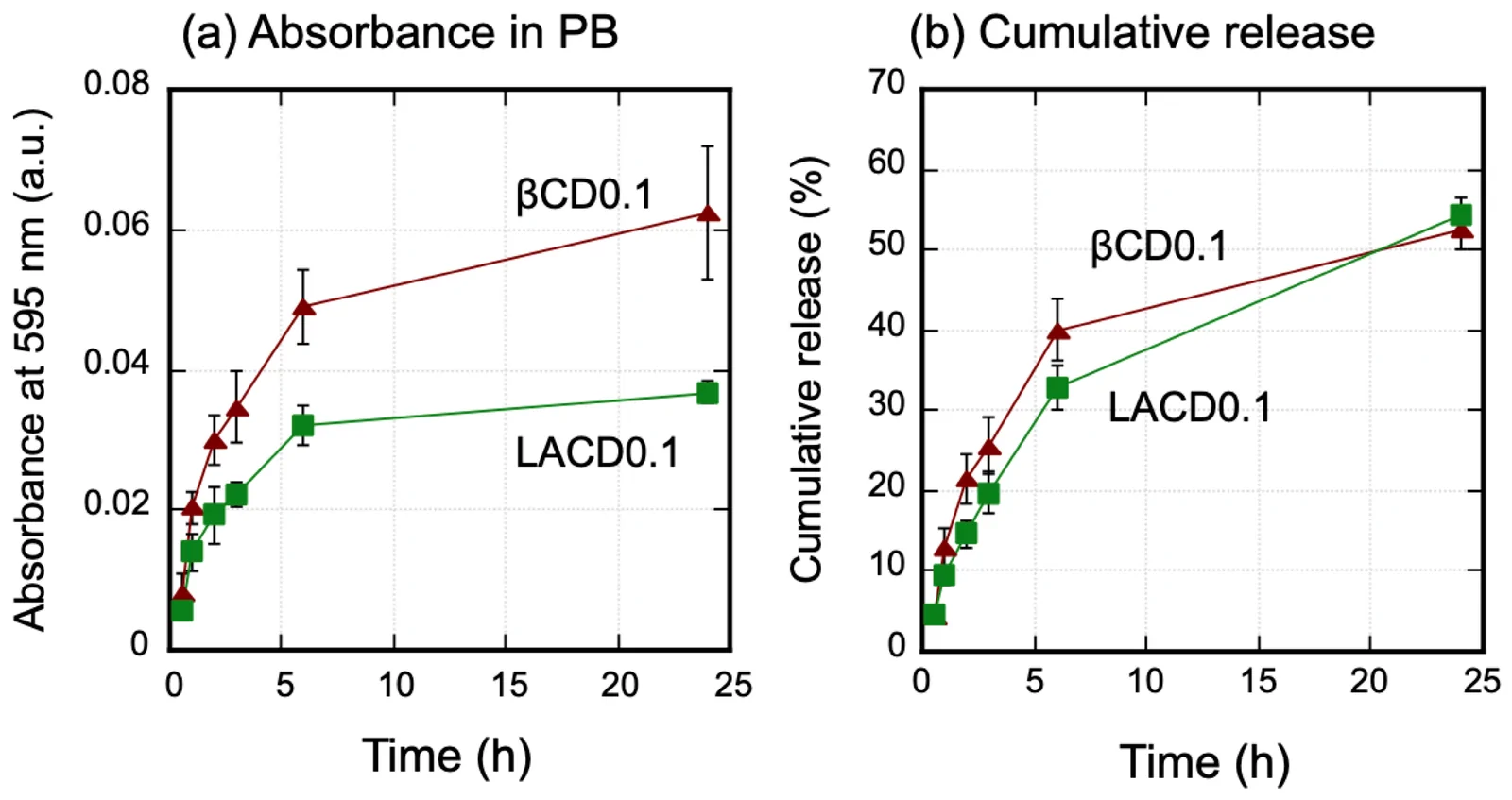
NR release from β-CD0.1–NR and LACD0.1–NR hydrogels in PB. (**a**) Absorbance at 595 nm of the aqueous release media from β-CD0.1–NR and LACD0.1–NR hydrogels in 0.1 M PB (pH 7.4). (**b**) Cumulative NR release determined after evaporation of the collected release samples and redissolution in methanol. Data are presented as mean ± SD (*n* = 3).

**Figure 10:**
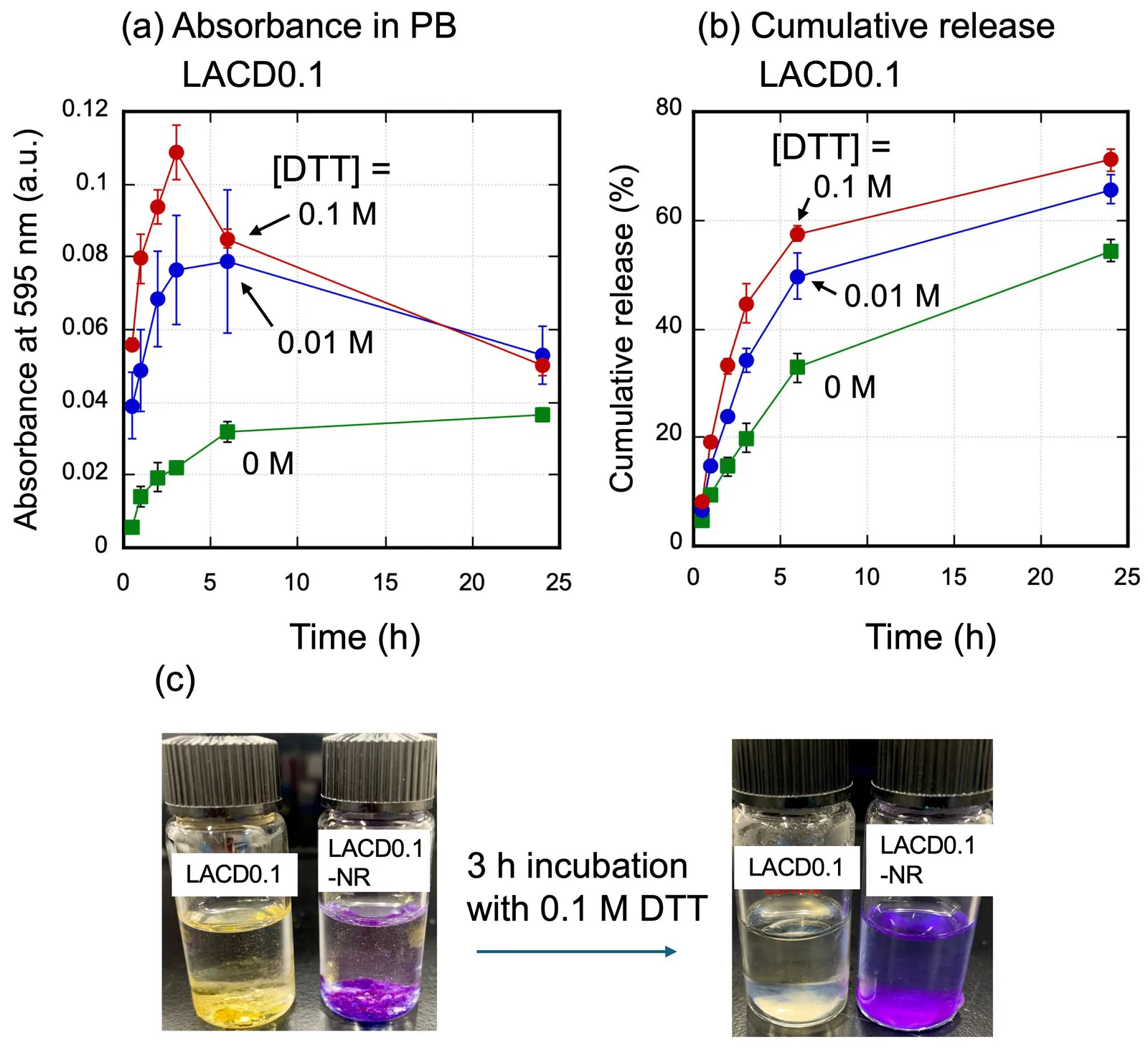
Effect of DTT on NR release from LACD0.1–NR hydrogels. (**a**) Absorbance at 595 nm of the aqueous release media from LACD0.1–NR hydrogels in PB alone (same as LACD0.1 in Figure 9a) or in PB containing 0.01 or 0.1 M DTT. (**b**) Cumulative NR release determined after evaporation of the collected release samples and redissolution in methanol under the same conditions. 0 M is the same as LACD0.1 in Figure 9b. (**c**) Photographs of blank LACD0.1 and NR-loaded LACD0.1 hydrogels during degradation in 0.1 M DTT from 0 to 3 h. Data are presented as mean ± SD (*n* = 3).

**Scheme 1:**
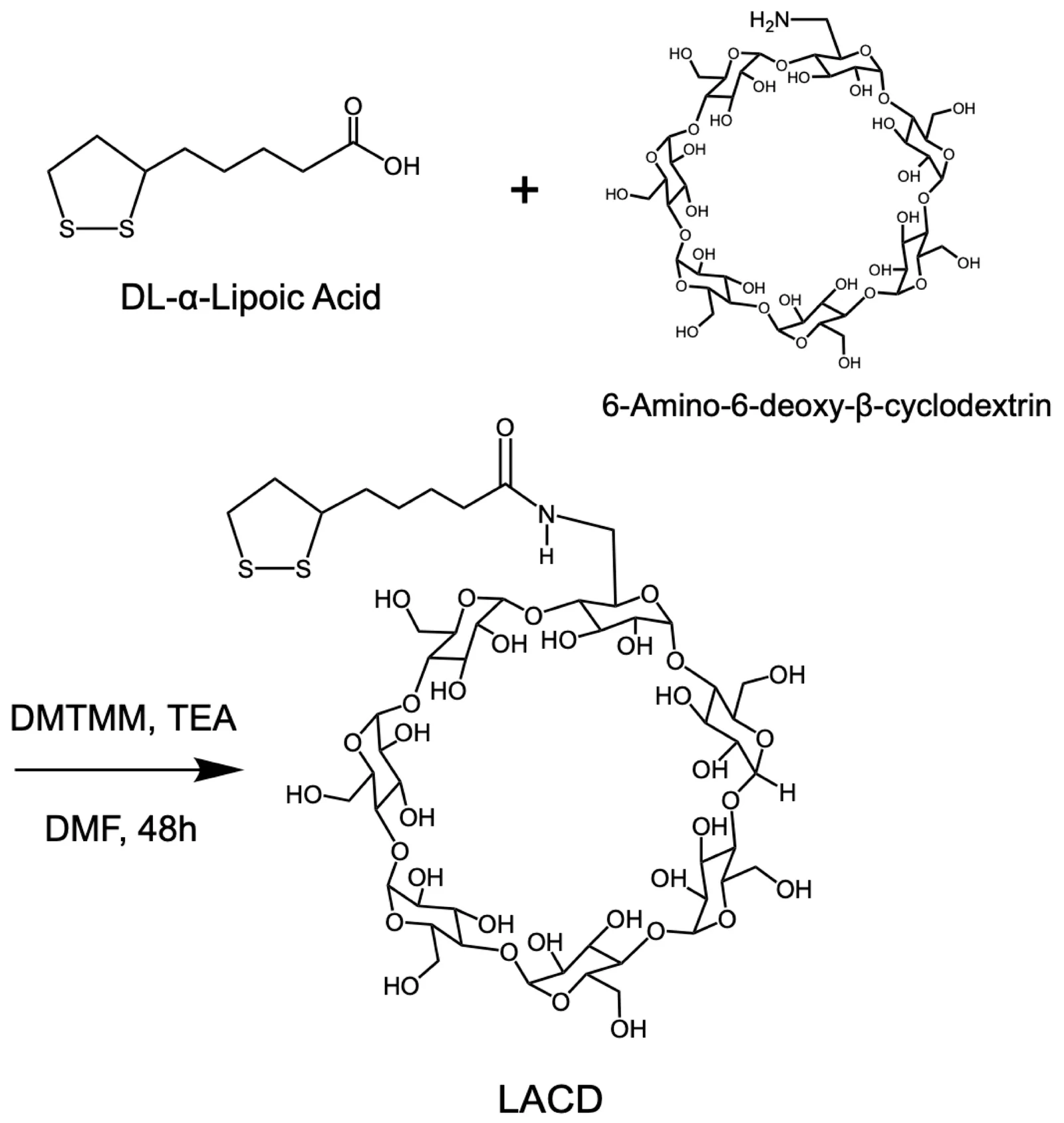
Synthetic route for 6-lipoylamido-β-cyclodextrin (LACD).

**Table 1:** Formulations of TA–LA and LACD-containing hydrogels.

Formulation	Tris Base	Tannic Acid (TA)	LACD	β-CD	α-Lipoic Acid (LA)	Water
TA0.2	200 mg (1.65 mmol)	200 mg (0.12 mmol)	−	−	1.0 g (4.85 mmol)	2 mL
TA0.3	300 mg (2.48 mmol)	300 mg (0.18 mmol)	−	−		
TA0.4	400 mg (3.30 mmol)	400 mg (0.24 mmol)	−	−		
LACD0.1	300 mg (2.48 mmol)	300 mg (0.18 mmol)	100 mg (0.08 mmol)	−		
LACD0.2	300 mg (2.48 mmol)	300 mg (0.18 mmol)	200 mg (0.15 mmol)	−		
β-CD0.1	300 mg (2.48 mmol)	300 mg (0.18 mmol)	−	100 mg (0.1 mmol)		
β-CD0.2	300 mg (2.48 mmol)	300 mg (0.18 mmol)	−	200 mg (0.18 mmol)		

**Table 2:** Polymerization-related ^1^H NMR signal comparison of TA–LA, β-CD0.1, and LACD0.1 hydrogels, mean ± SD (*n* = 3).

Sample	Poly(LA) Integral	Mono-LA Integral	Poly(LA) Molar Fraction (%)
TA–LA	3.0	4.1 ± 1.3	20 ± 4
β-CD0.1	3.0	6.1 ± 1.6	15 ± 3
LACD0.1	3.0	2.8 ± 0.3	27 ± 2

**Table 3:** Relative retention of CD H-1 signals in β-CD- and LACD-containing hydrogels after washing with water. Mean ± SD (*n* = 3).

Sample	CD Integral (Before Washing)	CD Integral (After Washing)	CD H-1 Signal Remaining (%)
β-CD0.1	0.9 ± 0.2	0.2 ± 0.1	21 ± 9
LACD0.1	0.6 ± 0.2	0.4 ± 0.2	72 ± 8

**Table 4:** NR encapsulation efficiency of TA–LA, β-CD, and LACD hydrogels (mean ± SD, *n* = 3).

Sample	Absorbance of Precursor Solution at 575 nm	NR Content in Hydrogel (μg/200 mg gel)	Encapsulation Efficiency (%)
β-CD0.1 gel	0.051 ± 0.004	19.0 ± 1.6	57 ± 5
LACD0.1 gel	0.082 ± 0.006	25.6 ± 1.5	77 ± 4
TA–LA gel	0.008 ± 0.002	2.0 ± 0.9	6 ± 3

**Table 5:** Early-stage release kinetics (0.5–3 h for zero-order k) and Korsmeyer–Peppas n.

Group	Zero-Order k (% h^−1^)	R^2^	Korsmeyer–Peppas n	R^2^
PB (LACD)	5.9	0.983	0.79	0.986
PB (β-CD)	8.2	0.933	0.85	0.947
DTT 0.01 M (LACD)	10.7	0.989	0.81	0.977
DTT 0.1 M (LACD)	14.3	0.986	0.94	0.985

## Data Availability

Data are contained within the article or Supplementary Materials.

## Supplementary Materials

The following supporting information can be downloaded at: https://www.mdpi.com/article/10.3390/gels12100868/s1. Figures S1–S3: Structural characterization of LACD by ^1^H NMR, ^13^C NMR, and ESI-HRMS. Figure S4: Aqueous interaction of LACD with Nile Red at different LACD concentrations. Figures S5 and S6: ADA-induced displacement of Nile Red from LACD. Figure S7: FTIR spectra. Figure S8: Inverted-vial photograph of gels. Figures S9–S13: Structural assignments and ^1^H NMR comparison of TA–LA, β-CD-containing, and LACD-containing hydrogels before and after washing. Figures S14–S18: UV–Vis quantification of Nile Red before gel formation, after hydrogel degradation, and in release samples. Figures S19–S23: Dilution stability of LACD–Nile Red in PB and DTT solutions. UV–Vis quantification of Nile Red before gel formation, after hydrogel degradation, and in release samples. Figure S24: Korsmeyer–Peppas analysis of Nile Red release. Figures S25 and S26: Nile Red calibration curve and UV–Vis spectra in methanol. Table S1: Dry mass ratios and gel fractions of hydrogels before and after washing. Table S2: Formulations of Nile Red-loaded hydrogels. Table S3: Preparation of Nile Red calibration standards in methanol.

## Abbreviations

The following abbreviations are used in this manuscript:

TA	Tannic acid
LA	Lipoic acid
β-CD	β-Cyclodextrin
LACD	6-Lipoylamido-β-cyclodextrin
NR	Nile Red
DTT	Dithiothreitol
PB	Phosphate buffer (0.1 M, pH7.4)
NMR	Nuclear magnetic resonance
ESI-HRMS	Electrospray ionization high-resolution mass spectrometry
FT-IR	Fourier-transform infrared spectrometry
SEM	Scanning electron microscopy
UV–Vis	Ultraviolet–visible spectroscopy
ADA	1-Adamantanecarboxylic acid
SD	Standard deviation
KP	Korsmeyer–Peppas
EE	Encapsulation efficiency
DMTMM	4-(4,6-Dimethoxy-1,3,5-triazin-2-yl)-4-methylmorpholinium chloride
TEA	Triethylamine
DMF	N,N-Dimethylformamide
