# Supplementary material for: Cyclodextrin-Containing Tannic Acid–Lipoic Acid Hydrogels for the Loading and Reduction-Responsive Release of Nile Red
Source: Gels. 2026 Sep 25;12(10):868. doi: 10.3390/gels12100868 (PMC13704123; doi:10.3390/gels12100868)
Supplement: Supplementary file 1 [file gels-12-00868-s001.zip › gels-4514114-supplementary.pdf]

Supplementary Materials

Supplementary Materials 1

S1. Characterization of LACD 3

S2. Gel fraction 6

S3. Aqueous Interaction of LACD with Nile Red 7

S4. ADA-Induced Displacement Ratio 7

S5. FTIR Spectra of the LACD–NR Inclusion Complex 9

S6. Hydrogel Formulations 10

S7. NMR integration and normalization 11

S8. UV–Vis Quantification of Nile Red in Hydrogels and Release Media 16

S9. Dilution stability experiment 20

S10. Calculation of cumulative release 23

S11. Statistical analysis 25

S12. Nile Red Calibration Curve in Methanol 27

List of Supplementary Figures

- **Figures S1–S3.** Structural characterization of LACD by ^{1}H NMR, ^{13}C NMR, and ESI-HRMS
- **Figure S4.** Aqueous interaction of LACD with Nile Red at different LACD concentrations.
- **Figures S5–S6.** ADA-induced displacement of Nile Red from LACD.
- **Figure S7.** FTIR spectra
- **Figure S8.** Inverted-vial photograph of gels.
- **Figures S9–S13.** Structural assignments and ^{1}H NMR comparison of TA–LA, β-CD-containing, and LACD-containing hydrogels before and after washing.
- **Figures S14–S18.** UV–Vis quantification of Nile Red before gel formation, after hydrogel degradation, and in release samples.

- **Figures S19–S23.** Dilution stability of LACD–Nile Red in PB and DTT solutions. UV–Vis quantification of Nile Red before gel formation, after hydrogel degradation, and in release samples.
- **Figure S24.** Korsmeyer–Peppas analysis of Nile Red release.
- **Figures S25–S26.** Nile Red calibration curve and UV–Vis spectra in methanol.

List of Supplementary Tables

- **Table S1.** Dry mass ratios and gel fractions of hydrogels before and after washing.
- **Table S2.** Formulations of Nile Red-loaded hydrogels.
- **Table S3.** Preparation of Nile Red calibration standards in methanol.

S1. Characterization of LACD

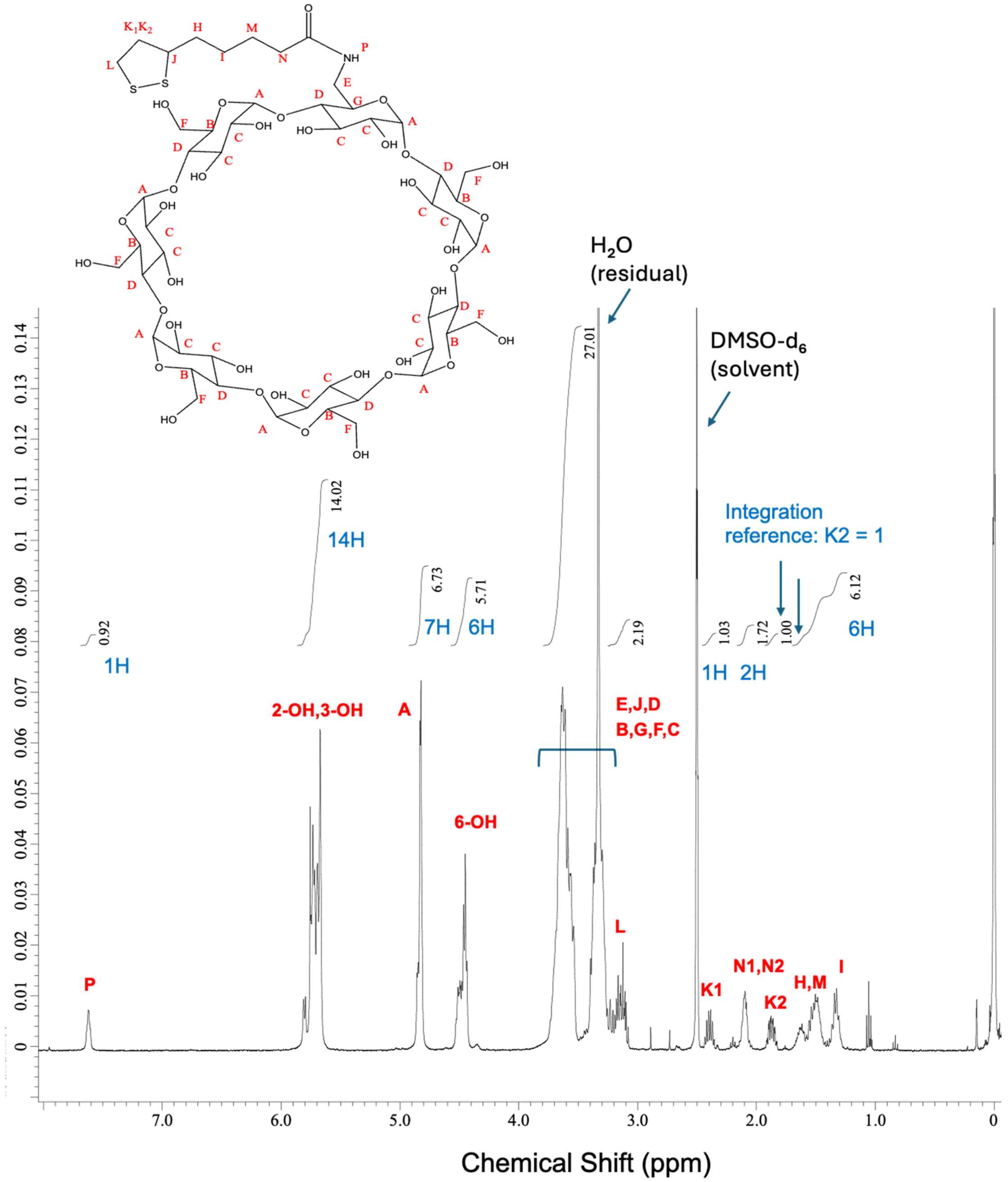

Figure S1. ^{1}H-NMR spectrum of LA-CD (DMSO-d$_6$, 400 MHz). The blue numbers indicate the theoretical number of protons for each signal. When the integral of K2 was set to 1, the integral values of the characteristic signals were in good agreement with the corresponding theoretical proton numbers.

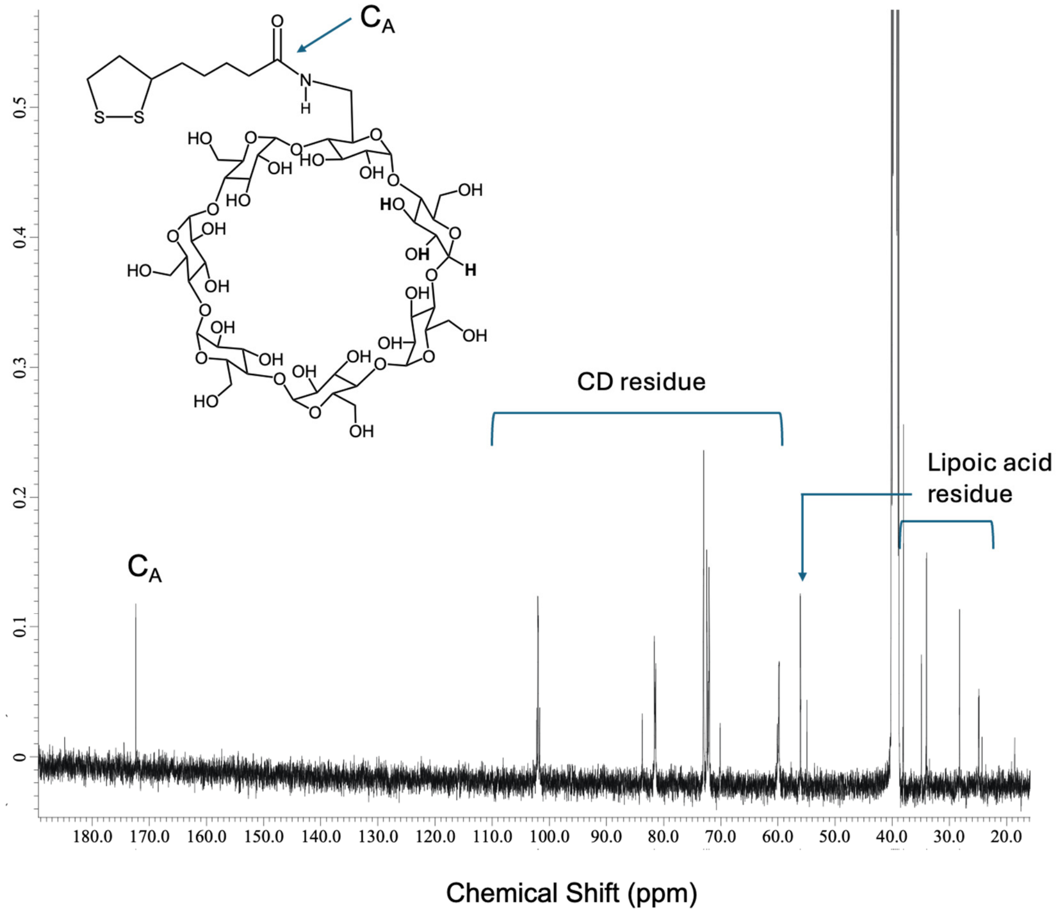

Figure S2. ^{13}C NMR spectrum of LA-CD (100 MHz DMSO-d_6)

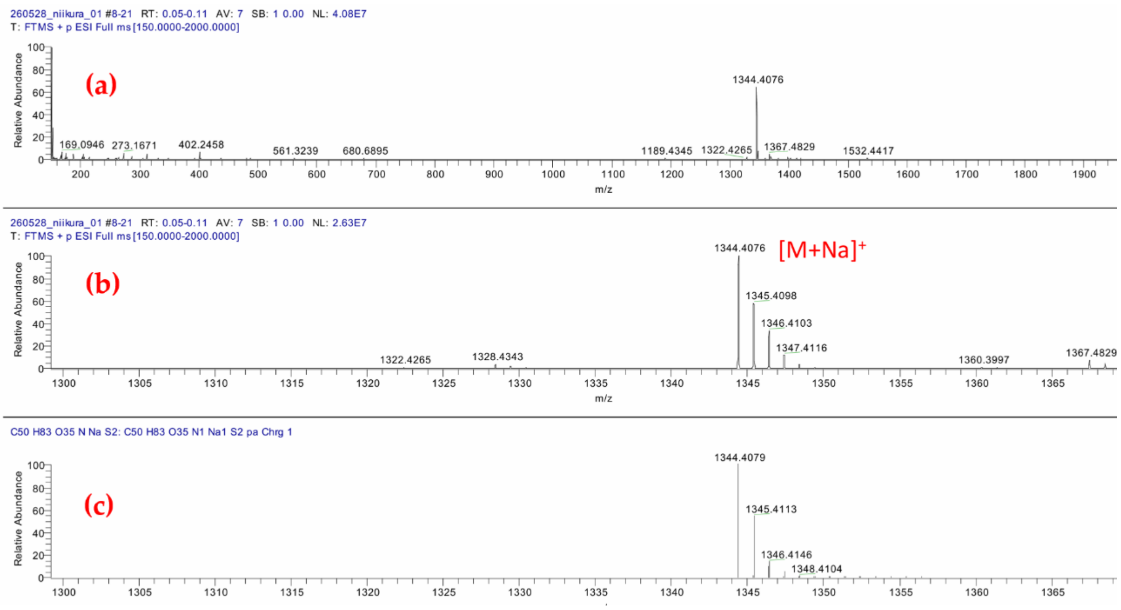

Figure S3. ESI-HRMS analysis of LACD. (a) Full-range experimental positive-ion ESI-HRMS spectrum. (b) Expanded view of the isotope cluster assigned to $[M+Na]^+$. (c) Calculated isotope pattern of $[M+Na]^+$. HRMS (ESI^+) *m/z*: $[M+Na]^+$ calcd for $C_{50}H_{83}NNaO_{35}S_2^+$, 1344.4079; found, 1344.4076.

S2. Gel fraction

Composition-matched hydrogel samples with an initial wet mass of 100 mg were assigned to before- and after-washing groups. The before-washing samples were freeze-dried directly, whereas the after-washing samples were immersed in deionized water for 24 h, rinsed with deionized water and ethanol, and then dried.

The gel fraction after washing was calculated as follows:

$$\text{Gel fraction (\%)} = \frac{\boldsymbol{W}_{\text{after}}}{\boldsymbol{W}_{\text{before}}} \times \mathbf{100} \qquad \text{Eq. (S1)}$$

Here, W_{before} and W_{after} refer to the dry weights of the hydrogel before and after the washing treatment, respectively.

Table S1. Dry mass ratios and gel fraction of hydrogels after washing treatment (mean ± SD, n = 3).

Formulation	Before washing dry mass ratio (%)	After washing dry mass ratio (%)	Gel fraction after washing (%)
TA–LA	48.2 ± 1.2	16.9 ± 2.5	38.2 ± 5.9
LACD 0.1	51.0 ± 0.8	18.5 ± 3.9	36.3 ± 7.7
LACD 0.2	52.5 ± 1.1	15.7 ± 3.6	29.9 ± 6.9
β-CD 0.1	49.5 ± 2.0	11.8 ± 4.6	23.8 ± 9.3
β-CD 0.2	51.5 ± 1.1	8.9 ± 1.6	17.3 ± 3.2

S3. Aqueous Interaction of LACD with Nile Red

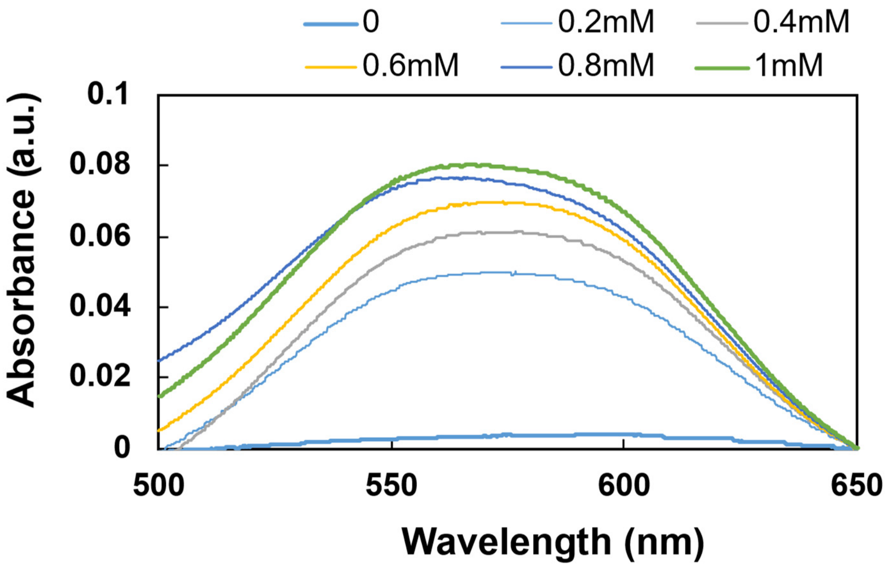

Figure S4. Mean UV–Vis absorption spectra of Nile Red in aqueous LACD solutions at different LACD concentrations over the wavelength range of 500–650 nm. Each spectrum represents the mean of three independent experiments (n = 3). The spectra were baseline-corrected by subtracting the absorbance at 650 nm.

S4. ADA-Induced Displacement Ratio

The apparent replacement ratio after ADA addition was calculated using the following equation:

$$\text{Displacement Rate (\%)} = \frac{\boldsymbol{A_0 - A_{ADA}}}{\boldsymbol{A_0}} \times \mathbf{100\%}$$

where A_0 is the absorbance at 575 nm before ADA addition, and A_{ADA} is the absorbance at 575 nm measured after adding ADA at a given concentration.

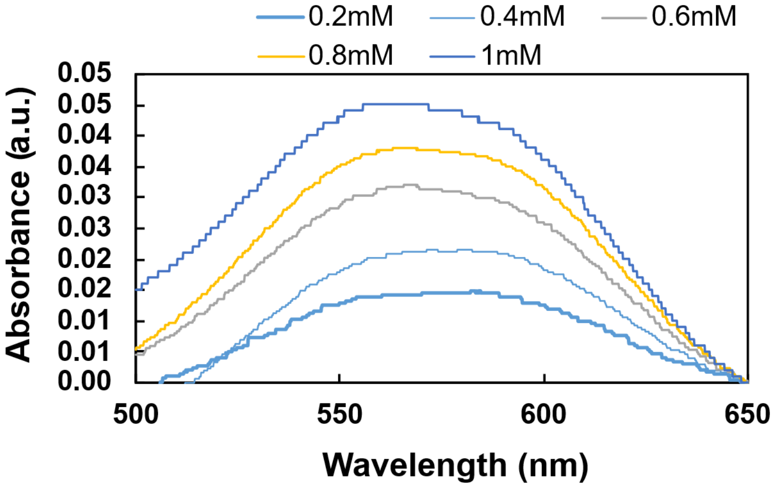

Figure S5. Mean UV–Vis absorption spectra of LACD–NR solutions after ADA addition at different LACD concentrations over the wavelength range of 500–650 nm. Each spectrum represents the mean of three independent experiments (n = 3). The spectra were baseline-corrected by subtracting the absorbance at 650 nm.

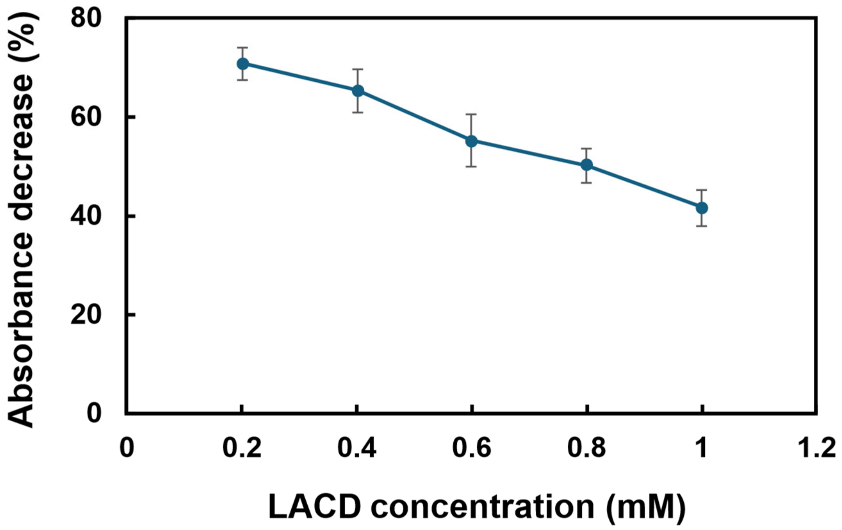

Figure S6. Apparent ADA-induced displacement ratio in LACD–NR solutions at different LACD concentrations. The displacement ratio was calculated from the decrease in absorbance at 575 nm after ADA addition. Data are presented as mean ± SD (n = 3).

S5. FTIR Spectra of the LACD–NR Inclusion Complex

The FTIR spectra were recorded using the KBr pellet method. The detected absorption bands were as follows: NR, 3410, 3059, 2967, 1622, 1494, 1407, 1350, 1184, 1114, 1079, 1017, 849, 780, 710, 661, and 419 cm^{-1}; LACD, 3306, 2925, 1653, 1583, 1429, 1369, 1333, 1251, 1204, 1157, 1031, 945, 755, 705, and 578 cm^{-1}; the LACD–NR physical mixture, 3310, 2925, 1624, 1584, 1407, 1311, 1256, 1031, and 579 cm^{-1}; and the LACD–NR inclusion complex, 3736, 3387, 2928, 1655, 1370, 1157, 1080, and 1030 cm^{-1}. The LACD/NR molar ratio was 8:1 in both the physical mixture and inclusion-complex samples.

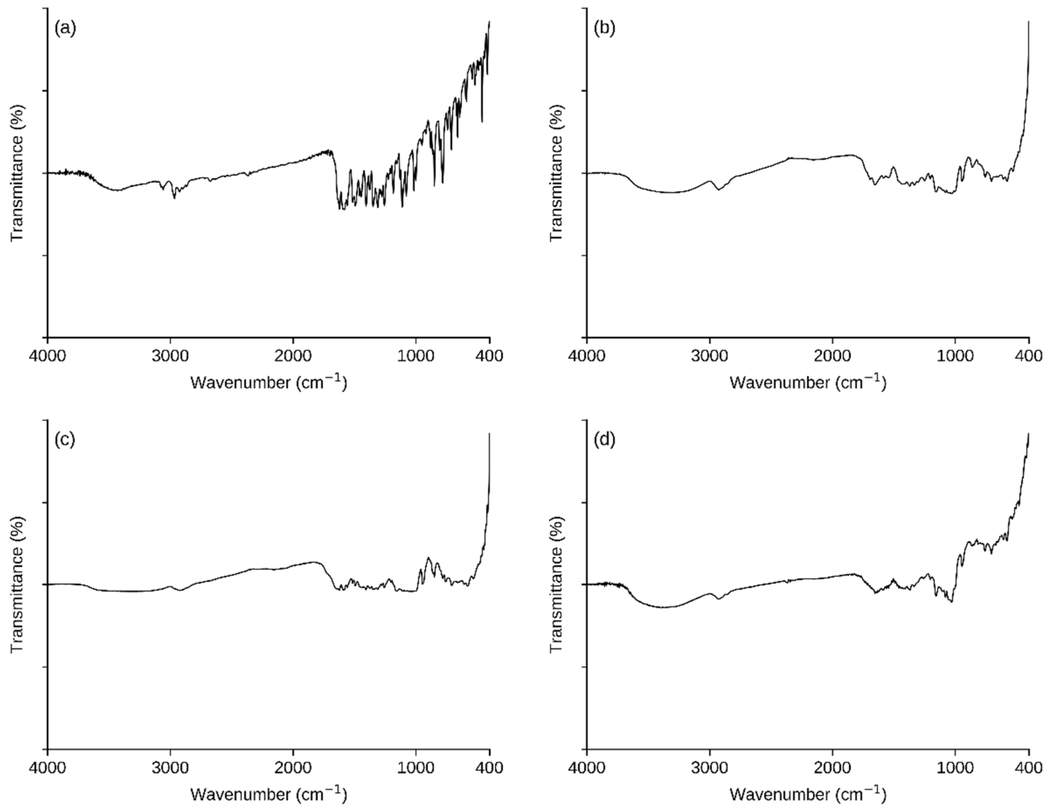

Figure S7. FTIR spectra of (a) NR, (b) LACD, (c) the LACD–NR physical mixture, and (d) the LACD–NR inclusion complex recorded using the KBr pellet method. The LACD/NR molar ratio was 8:1 in both (c) and (d).

S6. Hydrogel Formulations

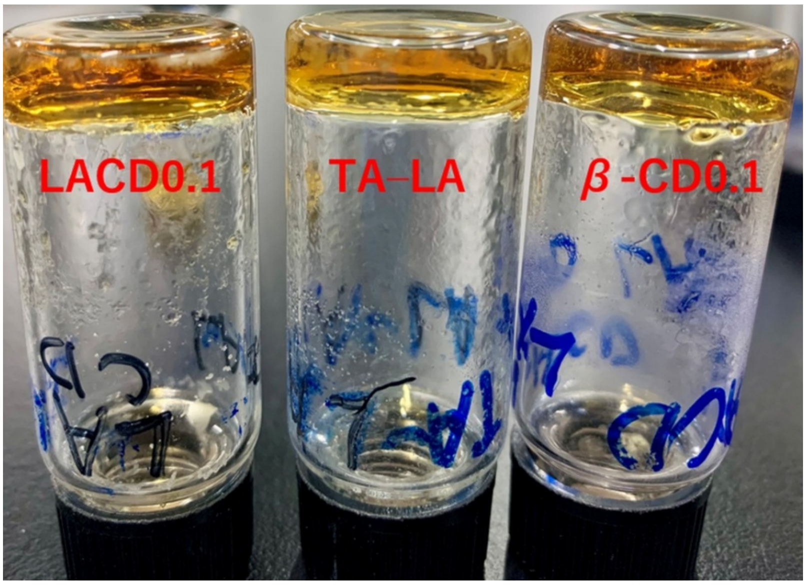

Figure S8. Inverted-vial photograph showing, from left to right, LACD0.1, TA-LA, and β-CD0.1hydrogels.

Table S2. Formulations of NR-loaded hydrogels

Formulation	CD component used for pre-association	Precursor solution	NR feed	Tris base	Tannic acid (TA)	α-Lipoic acid (LA)	Water for hydrogel formation
TA–LA–NR	—	Deionized water, 30 mL	0.10 mg	90 mg (0.74 mmol)	90 mg (0.05 mmol)	300 mg (1.46 mmol)	800 µL
β-CD0.1–NR	β-CD, 30 mg (0.026 mmol)	Deionized water, 30 mL	0.10 mg	90 mg (0.74 mmol)	90 mg (0.05 mmol)	300 mg (1.46 mmol)	800 µL
LACD0.1–NR	LACD, 30 mg (0.023 mmol)	Deionized water, 30 mL	0.10 mg	90 mg (0.74 mmol)	90 mg (0.05 mmol)	300 mg (1.46 mmol)	800 µL

S7. NMR integration and normalization

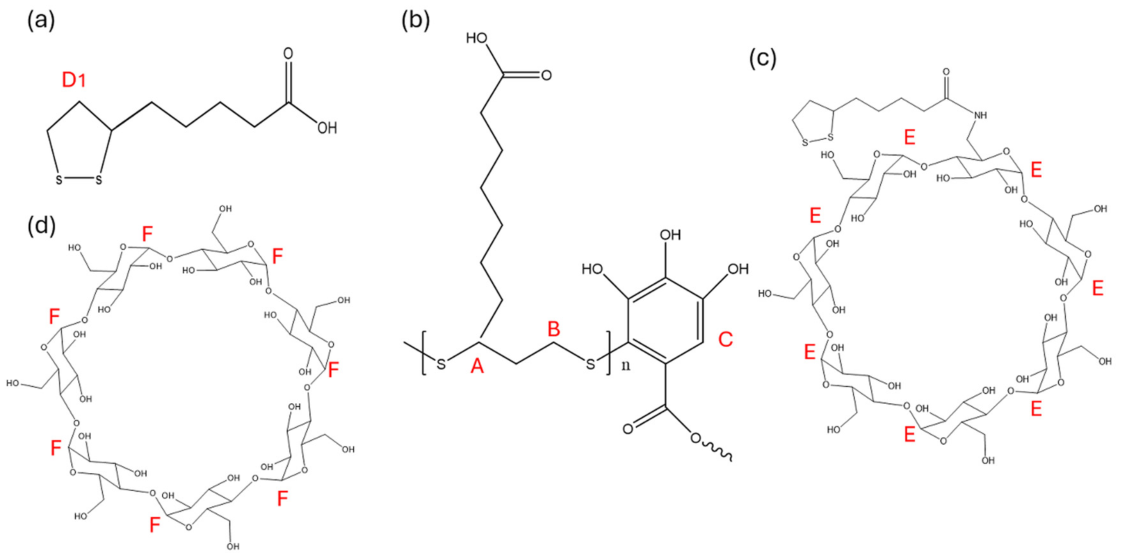

Figure S9. Schematic chemical structures of the hydrogel components and labelling of selected proton environments used for ^1H NMR peak assignment in DMSO-d_6: (a) lipoic acid monomer; (b) dynamic network formed by poly (lipoic acid) and tannic acid; (c) 6-lipoylamido-β-cyclodextrin (LACD); and (d) β-cyclodextrin (β-CD). D1 denotes one characteristic proton of the lipoic acid monomer; A and B denote selected proton environments in the poly (lipoic acid) chain, corresponding to a total of three protons; C denotes an aromatic proton of tannic acid; E denotes the anomeric H-1 protons of LACD; and F denotes the anomeric H-1 protons of β-CD.

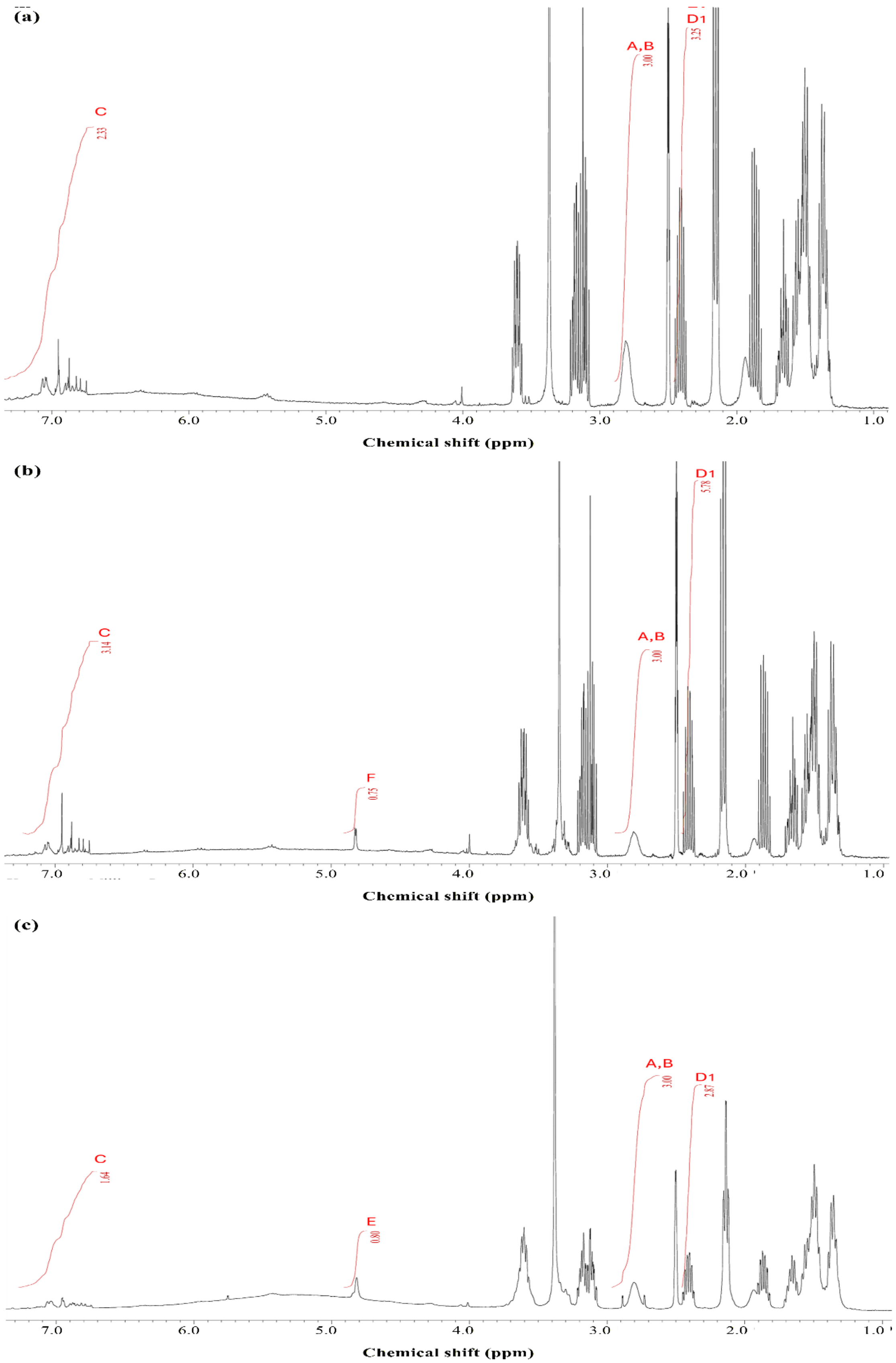

Figure S10. Representative ^{1}H NMR spectrum of the (a) TA-LA, (b) β-CD0.1, (c) LACD0.1 hydrogel before washing in DMSO-d_6 (400 MHz).

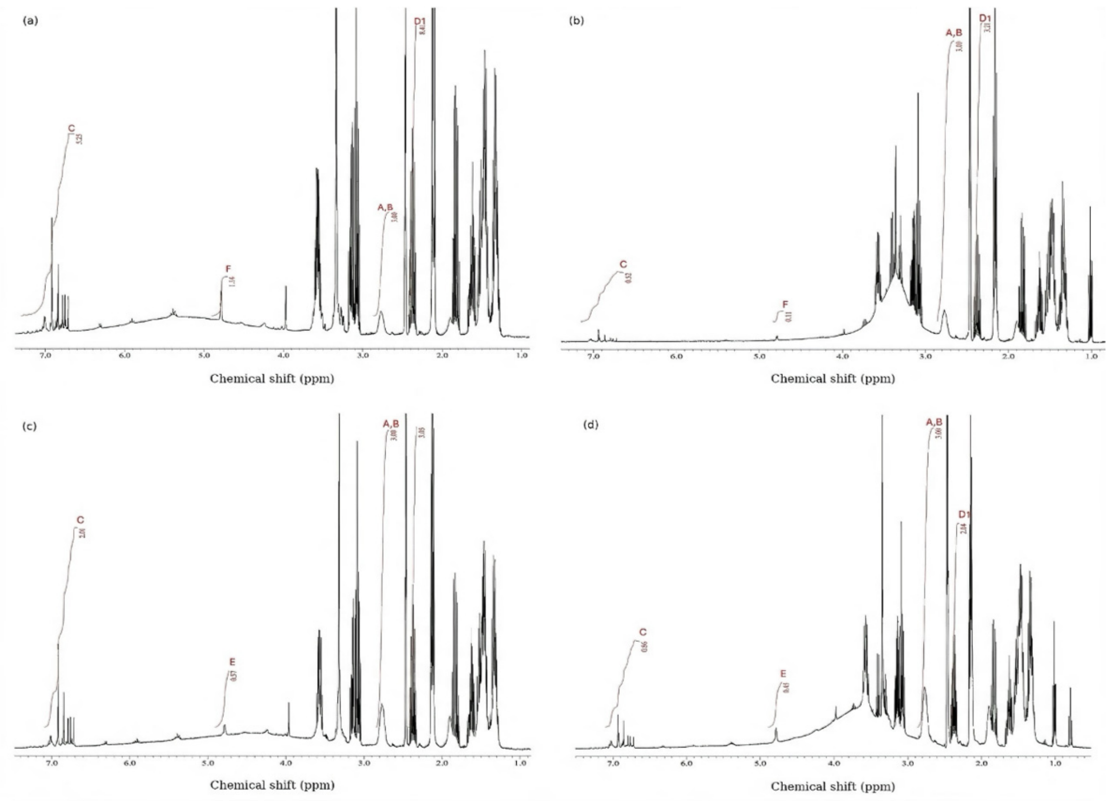

Figure S11. Representative ^1H NMR spectra of β-CD0.1 and LACD0.1 hydrogels before and after washing in DMSO-d_6 (400 MHz): (a) β-CD0.1 before washing, (b) β-CD0.1 after washing, (c) LACD0.1 before washing, and (d) LACD0.1 after washing. The spectra were normalized to the poly (LA)-related reference signal.

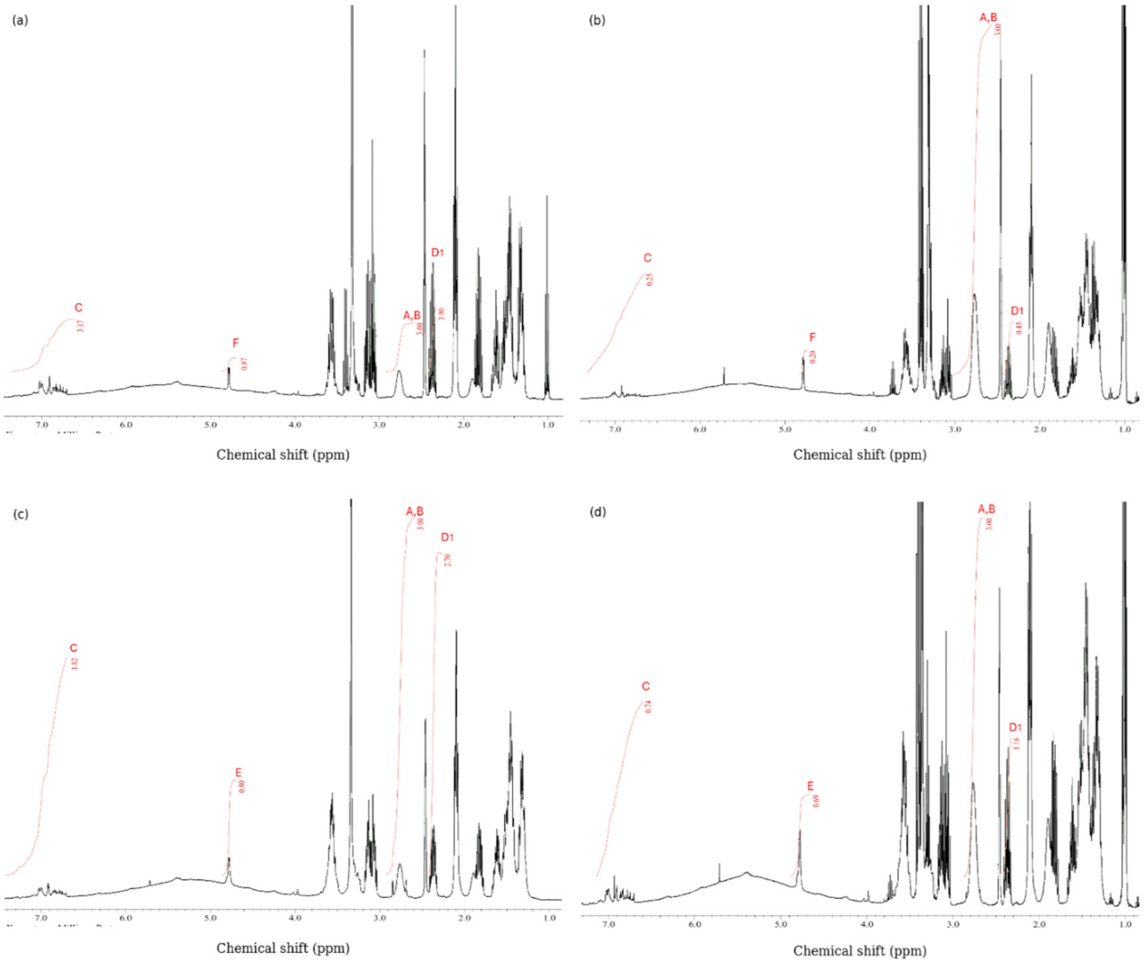

Figure S12. Representative ^{1}H NMR spectra of β-CD0.2 and LACD0.2 hydrogels before and after washing in DMSO-d_6 (400 MHz): (a) β-CD0.2 before washing, (b) β-CD0.2 after washing, (c) LACD0.2 before washing, and (d) LACD0.2 after washing. The spectra were normalized to the poly (LA)-related reference signal.

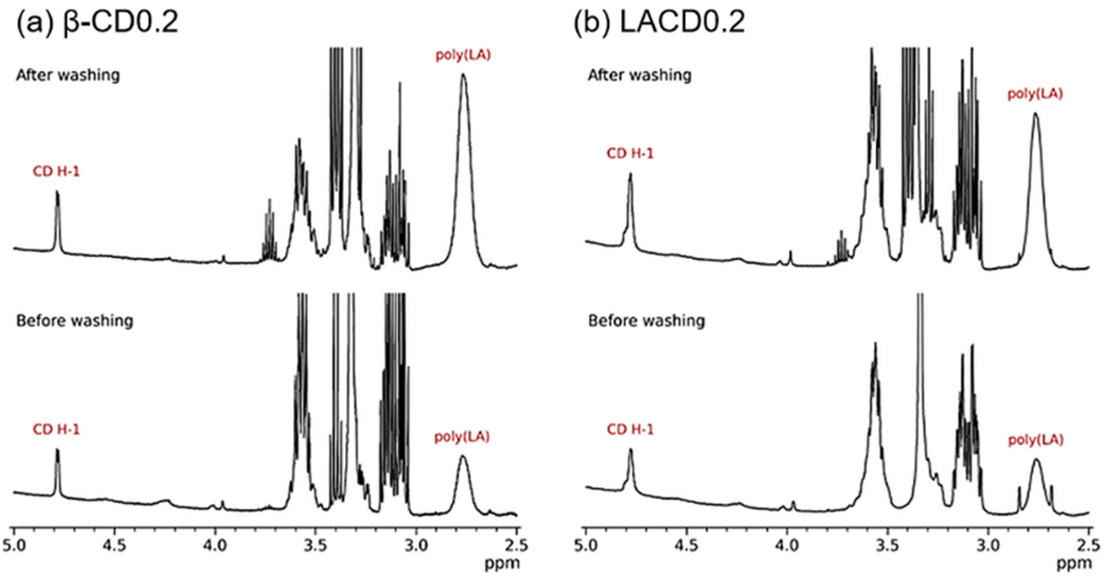

Figure S13. ^{1}H NMR spectra (2.5–5.0 ppm) of CD-containing TA–LA hydrogels before and after washing in DMSO-d_6 (400 MHz): (a) β-CD0.2, and (b) LACD0.2 hydrogels.

S8. UV–Vis Quantification of Nile Red in Hydrogels and Release Media

Freshly collected release samples were first analysed directly by UV–Vis spectroscopy over the wavelength range of 500–650 nm, and the absorbance at 595 nm was used to monitor the NR-related optical signal in the aqueous release medium. Release media collected at the corresponding time points from composition-matched blank hydrogels prepared without NR and incubated under the same conditions were used as baseline references. The same release samples were subsequently evaporated to dryness and redissolved in methanol for quantitative analysis. The spectra were baseline-corrected by subtracting the absorbance at 650 nm.

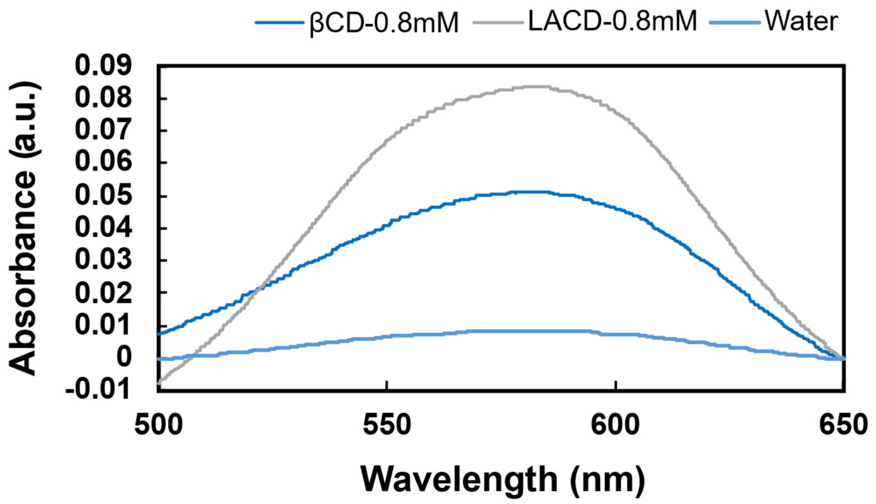

Figure S14. Mean UV–Vis absorption spectra of Nile Red in water, 0.8 mM β-CD, and 0.8 mM LACD solutions before hydrogel formation over the wavelength range of 500–650 nm. Each spectrum represents the mean of three independent experiments (n = 3).

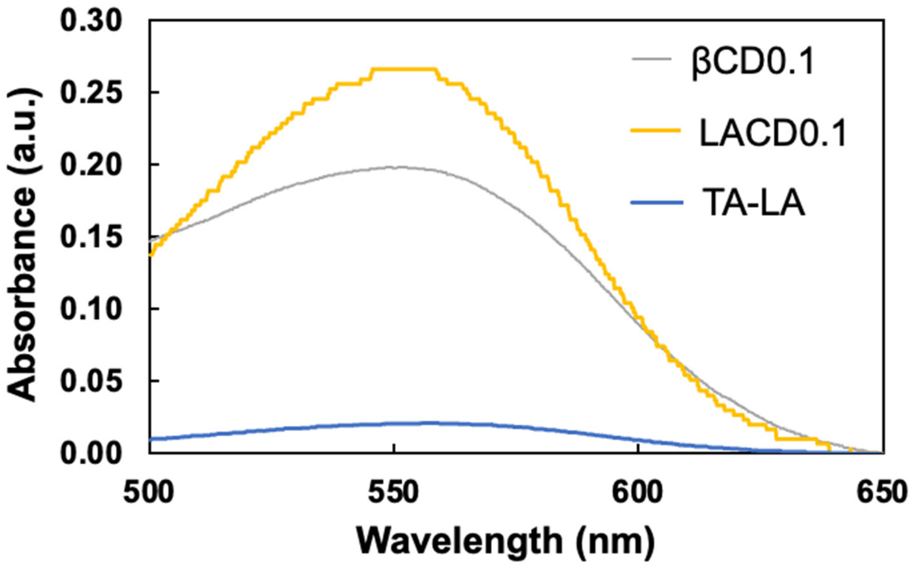

Figure S15. Mean UV–Vis absorption spectra of Nile Red recovered from fully degraded TA–LA (water), β-CD0.1, and LACD0.1 hydrogels after redissolution in methanol over the wavelength range of 500–650 nm. Each spectrum represents the mean of three independent experiments (n = 3). Error bands are omitted for clarity.

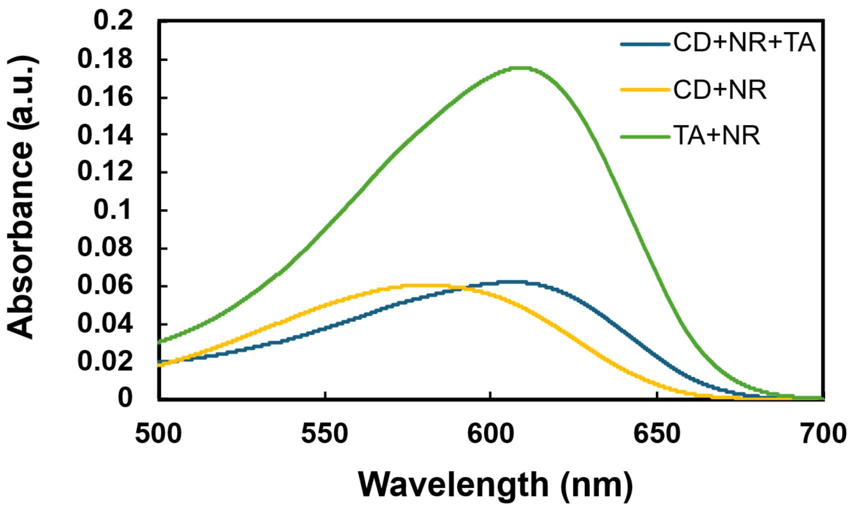

Figure S16. UV–Vis absorption spectra of Nile Red (NR) in water in the presence and absence of β-cyclodextrin (CD) and tannic acid (TA). [CD]=1.0 mg/mL, [NR]=1.9 μg/mL, [TA] =2.9mg/mL.

The samples for Fig. S16 were prepared at room temperature as follows. Stock solutions were prepared at 0.4 mg mL^{-1} NR in methanol, 1 mg mL^{-1} CD in water, and 100 mg mL^{-1} TA in water.

For CD + NR, 5 μL of the NR stock solution was added to 1 mL of the CD solution. NR was added in three portions, with sonication in a bath-type ultrasonic cleaner and vortex mixing after each addition to facilitate dissolution. Finally, 30 μL of water was added to make the total volume the same as that of the other samples.

For CD + NR + TA, 30 μL of the TA stock solution was added to 1 mL of the CD solution containing 5 μL of the NR stock solution (CD + NR).

For TA + NR, 30 μL of the TA stock solution was added to 1 mL of water, followed by the addition of 5 μL of the NR stock solution.

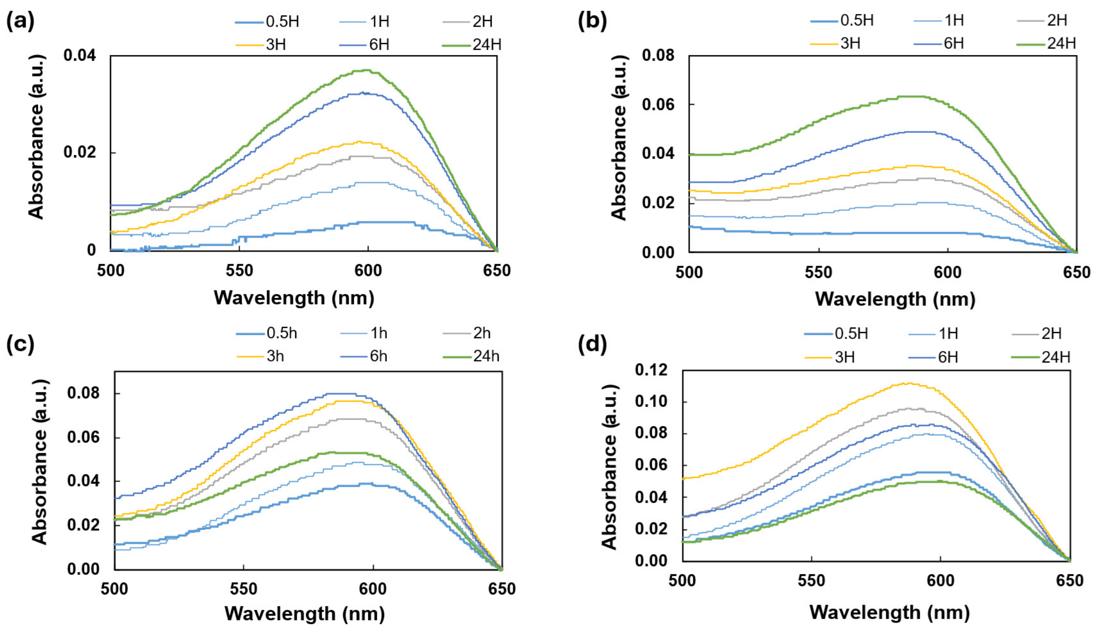

Figure S17. Mean UV–Vis absorption spectra of aqueous release media collected at different release times from (a) NR-loaded LACD0.1 hydrogels in 0.1 M PB (pH 7.4), (b) NR-loaded β-CD0.1 hydrogels in 0.1 M PB (pH 7.4), (c) NR-loaded LACD0.1 hydrogels in 0.01 M DTT, and (d) NR-loaded LACD0.1 hydrogels in 0.1 M DTT over the wavelength range of 500–650 nm. Each spectrum represents the mean of three independent experiments (n = 3). Error bands are omitted for clarity.

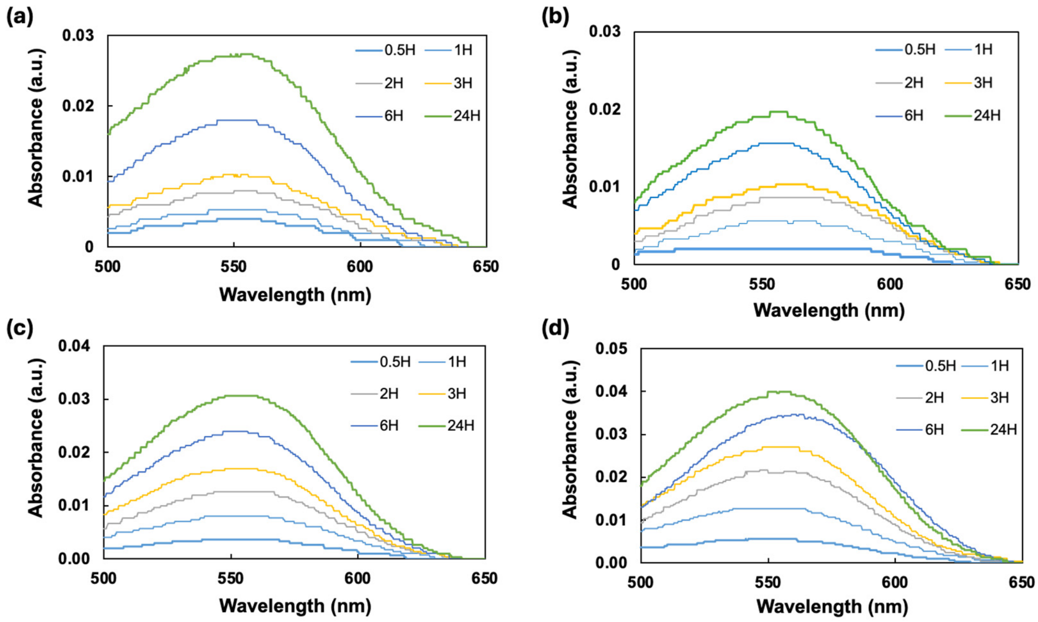

Figure S18. Mean UV–Vis absorption spectra of Nile Red recovered from release samples collected at different release times after evaporation and redissolution in methanol: (a) LACD0.1 hydrogels in 0.1 M PB (pH 7.4), (b) β-CD0.1 hydrogels in 0.1 M PB (pH 7.4), (c) LACD0.1 hydrogels in 0.01 M DTT, and (d) LACD0.1 hydrogels in 0.1 M DTT. Each spectrum represents the mean of three independent experiments (n = 3). Error bands are omitted for clarity.

S9. Dilution stability experiment

We investigated whether the presence of DTT in PB affected the aqueous absorbance of LACD–NR. Since CD–guest interactions are reversible, dilution can shift the equilibrium toward dissociation. Freshly prepared 0.8 mM LACD–NR solution (3 mL) was divided into three portions (1 mL each). Each portion was diluted with 4 mL of 0.1 M DTT, 0.01 M DTT, or 0.1 M PB (pH 7.4). UV–Vis absorption spectra were recorded from 500 to 650 nm at 0.5, 1, 2, 3, 6, and 24 h.

Absorbance decrease ratio was calculated using:

$$\text{Absorbance decrease ratio (\%)} = \frac{A_0 - A_t}{A_0} \times 100\%$$

where $A_0$and $A_t$are the absorbance values at 595 nm before dilution and at each time point after dilution, respectively. The spectra were baseline-corrected by subtracting the absorbance at 650 nm.

When we diluted 0.8 mM LACD–NR fivefold with PB, 0.01 M DTT, or 0.1 M DTT, the absorbance decreased similarly across all three groups, dropping by over 80% after 24 h compared with the initial post-dilution values (Fig. S8-S12). This suggests that the signal loss arose mainly from dilution-driven shifts in the LACD–NR equilibrium rather than a specific effect of DTT.

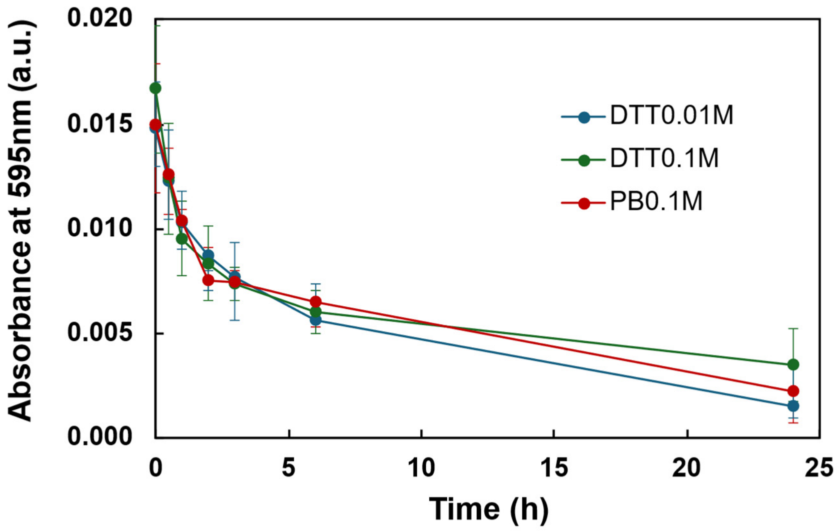

Figure S19. Time-dependent absorbance of LACD–NR solutions at 595 nm after fivefold dilution with PB, 0.01 M DTT, or 0.1 M DTT. Data are presented as mean ± SD (n = 3).

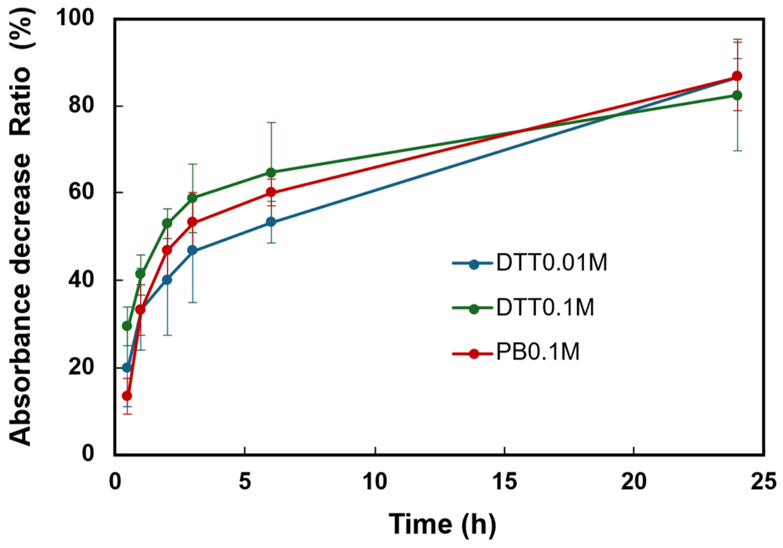

Figure S20. Relative decrease in absorbance of LACD–NR solutions after fivefold dilution with PB, 0.01 M DTT, or 0.1 M DTT. The absorbance measured immediately after dilution was used as the initial value. Data are presented as mean ± SD (n = 3).

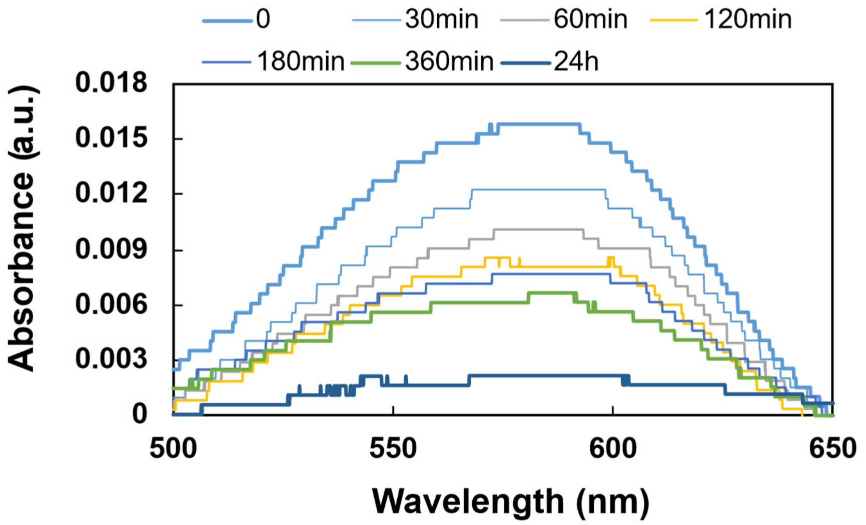

Figure S21. Mean UV–Vis absorption spectra of fivefold-diluted LACD–NR solutions in 0.01 M DTT over 500–650 nm during 24 h. Each spectrum represents the mean of three independent experiments (n = 3).

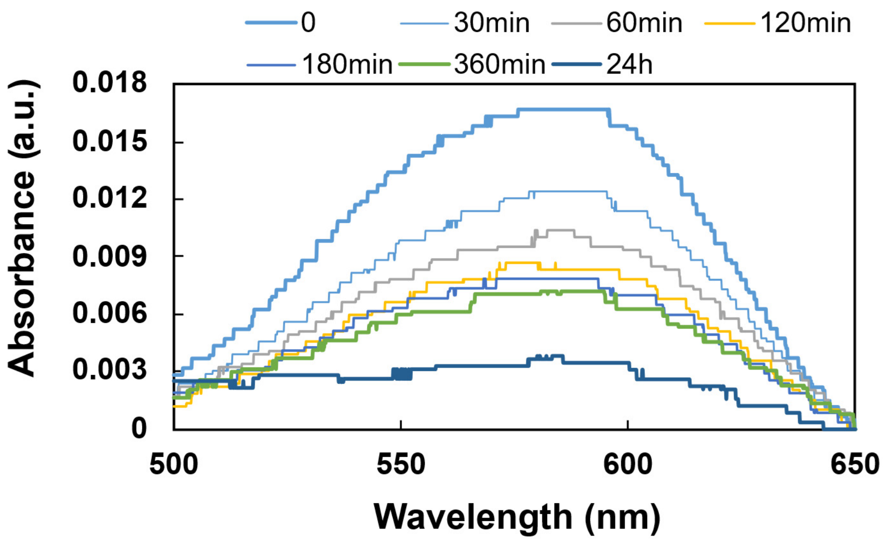

Figure S22. Mean UV–Vis absorption spectra of fivefold-diluted LACD–NR solutions in 0.1 M DTT over 500–650 nm during 24 h. Each spectrum represents the mean of three independent experiments (n = 3).

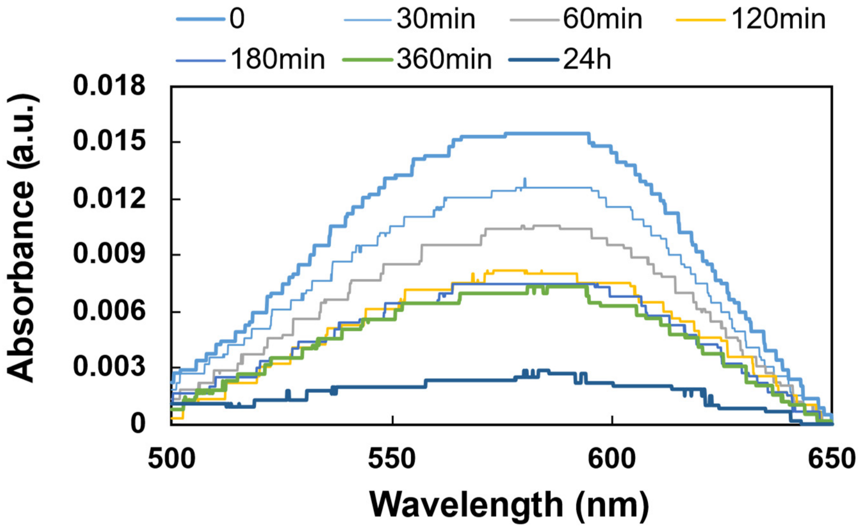

Figure S23. Mean UV–Vis absorption spectra of fivefold-diluted LACD–NR solutions in 0.1 M PB over 500–650 nm during 24 h. Each spectrum represents the mean of three independent experiments (n = 3).

S10. Calculation of cumulative release

At each sampling time, 200 μL of release medium was withdrawn from the total 2 mL medium and replaced with the same volume of fresh medium. Each

collected aliquot was evaporated to dryness, redissolved in 1 mL of methanol, and measured by UV–Vis spectroscopy. The blank-corrected absorbance was converted to NR concentration using the methanolic calibration curve:

$$A = 0.0208C$$
$$C = \frac{A}{0.0208}$$

where A is the blank-corrected absorbance and C is the NR concentration in methanol in μg mL^{-1}. Because each aliquot was redissolved in 1 mL of methanol, the NR amount in each collected aliquot was calculated as:

$$x_i = C_i \times 1 \text{ mL}$$

where x_i is the NR amount collected at sampling time i, in μg.

The cumulative amount released at time point n was corrected for repeated sampling. The NR removed at all previous time points was added to the amount present in the full 2 mL release medium at time point n:

$$M_{\text{released},n} = x_1 + x_2 + \cdots + x_{n-1} + 10x_n$$

The factor 10 was obtained from the ratio between the total release volume and the sampled volume:

$$\frac{2.0 \text{ mL}}{0.2 \text{ mL}} = 10$$

After the 24 h sample was collected, the remaining release medium and hydrogel were completely degraded, dried, and redissolved in 2 mL of methanol. The NR concentration in this final methanol solution was calculated from the same calibration curve and denoted as y. Therefore, the amount of NR remaining in the system was $2y$, and the total recoverable NR amount in the hydrogel was calculated as:

$$M_{\text{total}} = \sum_{i=1}^{6} x_i + 2y$$

The cumulative release percentage at time point n was then calculated as:

$$CR_n(\%) = \frac{x_1+x_2+\cdots+x_{n-1}+10x_n}{\sum_{i=1}^{6} x_i+2y} \times 100 \qquad \text{Eq. (S2)}$$

where:

- CR_n is the cumulative release percentage at time point n;
- x_i is the NR amount in the 200 μL aliquot collected at sampling time i;
- x_n is the NR amount in the aliquot collected at the current time point;
- $10x_n$ represents the NR amount present in the full 2 mL release medium at time point n;
- y is the NR concentration in the final 2 mL methanol solution after complete degradation of the remaining hydrogel;
- $2y$ is the NR amount remaining in the hydrogel and release medium after the final sampling;
- $\sum_{i=1}^{6} x_i$ is the total NR amount removed in the six collected aliquots.

This calculation corrects both the cumulative released amount and the total recovered NR amount for the NR removed during repeated sampling.

S11. Statistical analysis

All quantitative data are presented as mean ± standard deviation (SD) from three independent measurements unless otherwise stated.

Early-stage release behavior was analyzed by zero-order fitting over the first 3 h. The cumulative release percentage was plotted against time, and the slope of the linear regression was used as the apparent zero-order release constant (k_0):

$$CR = k_0 t + b$$

where CR is the cumulative release percentage at time t, k_0 is the apparent release constant (% h^{-1}), and b is the intercept. The fitting quality was evaluated using the coefficient of determination, R^2.

The release data were also fitted using the Korsmeyer-Peppas model.

$$\frac{M_t}{M_\infty} = k_{\mathrm{KP}}\, t^{\,n}$$

where $M_t/M_\infty$is the fractional release at time t, $k_{\mathrm{KP}}$is the Korsmeyer–Peppas release constant, and $n$is the release exponent. The equation was linearized as:

$$\log\left(\frac{M_t}{M_\infty}\right) = \log k_{\mathrm{KP}} + n \log t$$

The slope of the log–log plot gave the (n) value, and (R^2) showed how well the model fitted the data. Only data up to approximately 60% cumulative release were included in the Korsmeyer–Peppas fitting.

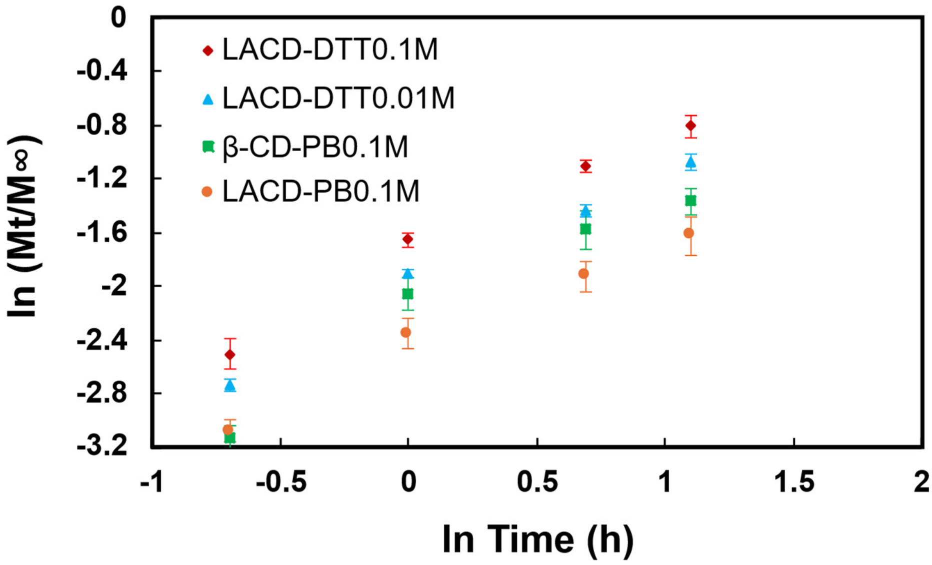

Figure S24. Linearized Korsmeyer–Peppas plots of Nile Red release from NR-loaded hydrogels under different release conditions: LACD0.1 in 0.1 M PB, β-CD0.1 in 0.1 M PB, LACD0.1 in 0.01 M DTT, and LACD0.1 in 0.1 M DTT. Although four data points are shown in the plot, for the Korsmeyer–Peppas fitting reported in Table 4, the initial release data up to 3 or 6 h, depending on the sample, were used, with the selected data points corresponding to a cumulative release of less than 60%.

S12. Nile Red Calibration Curve in Methanol

A stock solution of Nile Red in methanol (0.04 mg/mL, 0.13 mM) was diluted to six concentrations ranging from 0.0005 to 0.015 mg/mL (Table S5). UV–Vis spectra were recorded from 500 to 650 nm, and the absorbance at 555 nm was used for calibration.

The resulting calibration curve was fitted by linear regression according to:

$$A = 0.0208C \quad \text{Eq. (S3)}$$

where A is the absorbance at 555 nm and C is the NR concentration in μg/mL (R^2 = 0.999).

Table S3. Preparation of NR calibration standards in methanol.

Concentration (μg/mL)	Stock solution (μL)	Methanol (μL)
0.5	15	1185
1	30	1170
2	60	1140
4	120	1080
6	180	1020
8	240	960
12	360	840
15	450	750

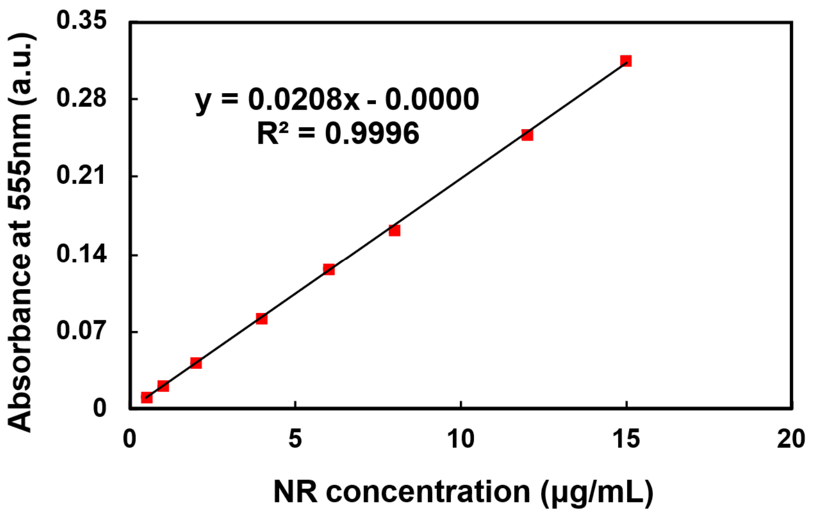

Figure S25. Nile Red calibration curve

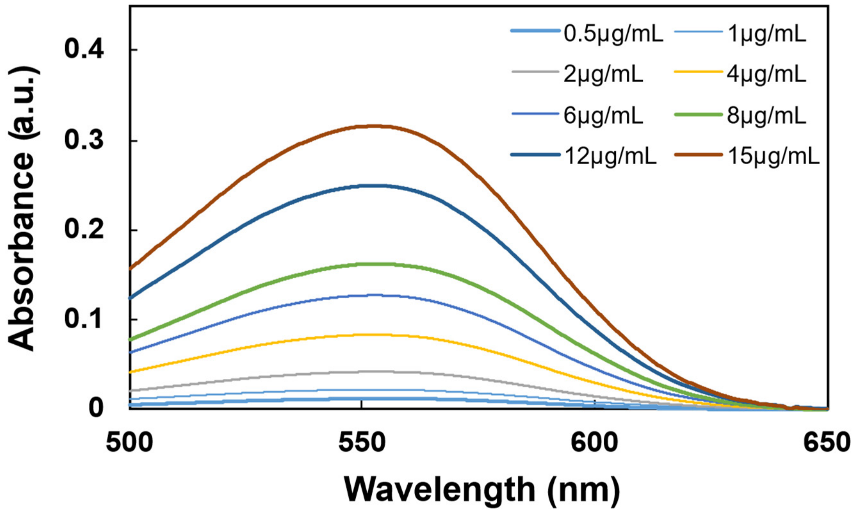

Figure S26. Mean UV–Vis absorption spectra of Nile Red in methanol at different concentrations over the wavelength range of 500–650 nm. Each spectrum represents the mean of three independent measurements (n = 3). The spectra were baseline-corrected by subtracting the absorbance at 650 nm.
